# BUB1 and CDK4/6 Dual Inhibition Increases Radiation Sensitivity in Glioblastoma, Lung Cancer, and Triple-Negative Breast Cancer

**DOI:** 10.3390/biomedicines14091940

**Published:** 2026-08-29

**Authors:** Shivani Thoidingjam, Sushmitha Sriramulu, Asya Haider Muratoglu, Rhea Hede-Sakhardande, Sunita Ghosh, Anthony J. Davis, Stephen L. Brown, Farzan Siddiqui, Benjamin Movsas, Corey Speers, Shyam Nyati

**Affiliations:** 1 Department of Radiation Oncology, Henry Ford Health-Cancer, Detroit, MI 48202, USAsbrown1@hfhs.org (S.L.B.);; 2 Henry Ford Health + Michigan State University Health Sciences, Detroit, MI 48202, USA; 3 Department of Radiology, Michigan State University, East Lansing, MI 48824, USA; 4 Department of Public Health Sciences, Henry Ford Health, Detroit, MI 48202, USA; 5 Department of Radiation Oncology, University of Texas Southwestern, Dallas, TX 75390, USA; 6 Department of Radiation Oncology, University of Alabama, Birmingham, AL 35233, USA

**Keywords:** radiotherapy, BUB1, CDK4/6, DNA repair, HR, NHEJ

## Abstract

**Background:** Solid tumors including glioblastoma (GBM), lung cancer (LC), and triple-negative breast cancer (TNBC) exhibit marked radioresistance driven by dysregulated cell-cycle control and genomic instability. Although CDK4/6 inhibitors suppress tumor proliferation, their radiosensitizing capacity is limited by persistent DNA repair. BUB1, a mitotic checkpoint kinase overexpressed in aggressive cancers, has emerged as a regulator of DNA damage signaling. We tested whether co-targeting BUB1 and CDK4/6 enhances radiosensitivity across solid tumors. **Methods:** GBM, LC, and TNBC cell lines were treated with BUB1 inhibitor BAY1816032, CDK4/6 inhibitors ribociclib and abemaciclib, and radiation. Proliferation, clonogenic survival, immunoblotting, and combination index analyses assessed cytotoxicity and synergy. CDK4/6-resistant models were generated to examine resistance. In vivo efficacy was evaluated using SUM159 xenografts. DNA damage and homologous recombination repair were measured by gH2AX, RAD51, RPA, BrdU foci and comet assay. The resection branchpoint was assessed by phospho-RPA, with ATR inhibition and BLM or EXO1 depletion testing resection dependence. **Results:** BUB1 inhibition increased cytotoxicity in vitro and improved therapeutic response in vivo. Combined BUB1 and CDK4/6 inhibition showed strong synergy (C.I. < 1) and enhanced radiosensitization in RB+ models. CDK4/6-resistant cells displayed increased BUB1 expression, and BUB1 inhibition partially restored sensitivity. Mechanistically, dual inhibition intensified homologous recombination defects, marked by persistent gH2AX and altered RAD51, RPA, and BrdU dynamics, consistent with sustained single stranded DNA and impaired HR repair. Persistent RPA32 Ser33 phosphorylation reflects ATR-dependent resection that requires BLM and EXO1 at later stages, supporting sustained resection and unresolved repair leading to increased cell death. **Conclusions:** Dual inhibition of BUB1 and CDK4/6 represents a promising therapeutic strategy for enhancing radiosensitivity in GBM, lung cancer, and TNBC, particularly in Rb-intact settings.

## 1. Introduction

Solid cancers such as glioblastoma multiforme (GBM), some aggressive lung cancers such as non-small cell lung cancer (NSCLC), and triple-negative breast cancer (TNBC) are associated with poor prognosis and limited treatment options [1,2,3,4]. Globally, lung cancer remains the most frequently diagnosed cancer and the leading cause of cancer-related mortality, accounting for approximately 2.6 million new cases and 1.9 million deaths in 2024 [5]. TNBC represents approximately 10–15% of all breast cancers and is characterized by an aggressive clinical course and a lack of approved hormone receptor or HER2-targeted therapies [3,6], while GBM remains the most common and aggressive primary malignant brain tumor in adults with a median survival of 14 months despite standard-of-care treatment [7,8,9]. Traditional therapies like chemotherapy and radiation often fail to provide long-term benefits due to the development of resistance and systemic toxicity [2,10,11,12]. While these cancers differ clinically, they share common defects in cell-cycle regulation and DNA damage response pathways, providing a strong biological rationale for testing unified radiosensitization approaches. To address the critical need for improved therapeutic options, specific molecular pathways implicated in cancer progression are being explored. Among these, BUB1 and CDK4/6 have emerged as promising targets due to their essential roles in regulating cell-cycle progression and genomic stability [13,14,15,16,17,18,19,20].

Mitotic Ser/Thr kinase BUB1, originally described as Budding Uninhibited by Benzimidazoles-1, is a core regulator of the mitotic checkpoint required for accurate chromosome segregation during cell division [16]. BUB1 dysregulation has been reported across multiple solid cancers [21,22,23], and its overexpression correlates with poor prognosis in cancers including GBM, NSCLC and TNBC [20,24,25,26,27,28,29].

Cyclin-dependent kinases 4 and 6 (CDK4/6) are critical regulators of the G1/S phase transition and are frequently overactivated in cancer-promoting unchecked proliferation [30,31]. Aberrant CDK4/6 signaling contributes to tumor growth and therapeutic resistance in GBM, LC and TNBC [32,33,34]. CDK4/6 inhibitors have demonstrated strong preclinical and clinical activity, resulting in approval for select malignancies [35]. In glioblastoma models, agents including palbociclib, abemaciclib, and ribociclib show antitumor activity and brain penetration [36,37,38,39,40]. However, despite central nervous system exposure, abemaciclib monotherapy has not produced durable clinical benefit in glioblastoma, underscoring the need for rational combination strategies [38,41]. While CDK4/6 inhibition is established in hormone receptor positive breast cancer, its role in TNBC, NSCLC, and glioblastoma remains under investigation, motivating combination approaches that address complementary cell-cycle and DNA repair vulnerabilities [33].

BUB1 inhibition disrupts spindle checkpoint function and induces mitotic catastrophe in solid tumors [42,43,44], while CDK4/6 inhibition enforces G1 arrest through suppression of DNA replication programs and RB phosphorylation [30,45]. Together, these pathways represent complementary targets in cell-cycle control. Building on prior evidence supporting BUB1 and CDK4/6 inhibitors in solid cancers [32,37,38,39,40,46], we investigated the effects of combined BUB1 and CDK4/6 inhibition, with or without radiation therapy, in glioma, NSCLC, and TNBC models in vitro and in vivo.

## 2. Materials and Methods

### 2.1. Correlation Analysis

Correlation analyses were performed using cBioPortal (cBioPortal for Cancer Genomics) to evaluate the relationship between BUB1 expression and genes involved in the CDK4/6-RB signaling pathway in breast cancer. The breast cancer (METABRIC, Nature 2012 & Nat Commun 2016) dataset with 2509 samples was used for all analyses. Correlations between BUB1 mRNA expression and CDK4, CDK6, RB1, CCND1, CDK2, E2F1, E2F2, E2F3, and E2F7 were assessed to investigate potential associations between BUB1 expression and cell-cycle regulatory pathways.

### 2.2. Cell Culture

Cells with different driver mutations were used in this study (Table 1). GBM (U251, A172, U87, 9L), NSCLC (A549, H1975, H596), TNBC (MDA-MB-231, BT-549, HCC1937) and breast epithelial MCF10A cell lines were obtained from ATCC. SUM159 was acquired from Sofia Merajver (University of Michigan, Ann Arbor). Cell lines were cultured in DMEM (Catalog #30-2002, ATCC), RPMI-1640 (Catalog #30-2001, ATCC) or HAM’S F-12 media (Catalog #31765035, Thermo Fisher Scientific, Waltham, MA, USA) supplemented with 5% FBS, 10 mM HEPES, 1 μg/mL Hydrocortisone, and 6 μg/mL Insulin. 1% Penicillin–Streptomycin (5000 U/mL) (Catalog #15070063, Thermo Fisher Scientific, Waltham, MA, USA) was added to all media. Cells were maintained at 37 °C with 5% CO_2_ and were regularly tested for Mycoplasma contamination using the MycoAlert PLUS kit (Catalog #LT07-705, Lonza, Basel, Switzerland).

### 2.3. Drug Treatment

BUB1 inhibitor BAY1816032 (Catalog #CT-BAY181, Chemietek, Indianapolis, IN, USA), CDK4/6 inhibitors ribociclib and abemaciclib (#HY-15777 and #LY2835219, Medchem Express, Monmouth Junction, NJ, USA), and ATR inhibitor (BAY1895344/Elimusertib, # S8666) were obtained from Selleck Chemicals, Houston, TX, USA. Stock solutions of BAY1816032 (20 mM), ribociclib (10 mM), abemaciclib (5 mM) and BAY1895344 (10 mM) were made in DMSO, aliquoted and stored at −80 °C. Working solutions with varying drug concentrations in culture media were freshly prepared to maintain drug stability and activity during assays. The final DMSO concentration in control groups was 0.1% (*v*/*v*).

### 2.4. Cell Viability Assay

Cells were seeded at 1000 cells per well in 96-well plates. After 24 h, cells were treated with BAY1816032, ribociclib or abemaciclib at micromolar concentrations. For drug combination experiments, cells were treated with BUB1i + ribociclib or BUB1i + abemaciclib concurrently or sequentially. After 72 h of drug treatment, cell proliferation was assessed using the AlamarBlue^®^ Cell Viability Reagent (Catalog #DAL1100, Thermo Fisher Scientific) according to the manufacturer’s protocol. AlamarBlue^®^ Cell Viability Reagent diluted 1:10 in fresh culture medium was added to each well (200 μL/well). Cells were incubated for 3–4 h at 37 °C, after which absorbance was measured. Data were analyzed using GraphPad Prism v10, and *p*-values were calculated to determine the significance.

### 2.5. BUB1 CRISPR Knockout Cell Lines

To generate BUB1 knockout cell lines using CRISPR/Cas9 technology, we targeted two different exons of the BUB1 gene (exon 2 and exon 3). Detailed protocol of BUB1 knockout clone generation, gRNA sequences, primer sequences for PCR, and Sanger sequencing information are provided in our previous study [20].

### 2.6. Radiation

A CIX3 cabinet irradiator (Xstrahl Inc., Suwanee, GA, USA) equipped with a 320 kV metal ceramic X-ray tube operating at 10 mA was used to deliver radiation. Beam hardening was achieved using a 1 mm copper filter. Cell monolayers cultured in 6-well plates were irradiated with doses of 2 or 4 Gy at a dose rate of approximately 2 Gy/min. Quality assurance procedures included routine verification with a calibrated, certified small-field electrometer and the regular use of EBT3 Gafchromic film (Ashland Specialty Ingredients, Wilmington, DE, USA) to confirm accurate radiation delivery.

### 2.7. Clonogenic Assay

The clonogenic cell survival assay was used to assess the radiation sensitization by BUB1 inhibitor in combination with CDK4/6i. Cells were seeded in 6-well plates at varying densities in triplicate (cell numbers shown in Table S1), then treated 24 h later with different concentrations of BAY1816032, ribociclib, or abemaciclib administered alone, in combination, or in sequential regimens. Cells were irradiated with 2 or 4 Gy either 1 or 24 h after drug treatment and allowed to grow for 1–3 weeks until colonies (>50 cells) formed. Colonies were fixed, stained, and counted to determine survival fractions (SFs) and radiation enhancement ratios (rERs) as previously described [19,20]. Radiation enhancement ratios (rERs) were calculated from clonogenic survival curves using Microsoft Excel. The rER was defined as the ratio of the radiation dose required to achieve the same level of cell killing in vehicle-treated versus inhibitor-treated cells. An rER > 1 was considered indicative of radiosensitization, whereas an rER < 1 indicated radioprotection or radiation resistance.

### 2.8. Establishment of CDK4/6i Resistant Cell Lines

Cell lines resistant to the CDK4/6 inhibitors ribociclib and abemaciclib were generated using a stepwise exposure method. Initially, cells were cultured with ribociclib and abemaciclib at their IC_30_ concentrations for one week, followed by a week at their IC_50_ concentrations. Subsequently, the concentrations were raised to IC_70_ for a month and then maintained in IC_70_ for chemoresistant BT549 cells or increased to IC_90_ for SUM159 chemoresistant cells for maintenance [47,48].

### 2.9. In Vivo Studies

Eight-week-old female Fox Chase SCID mice (Charles River Laboratories, Wilmington, MA, USA) were acclimatized for 2–3 weeks before study initiation. Orthotopic tumors were established by bilateral injection of SUM159 cells into the fourth mammary fat pads. All procedures followed HFH-IACUC protocol #00001490 (approval date: 21 July 2023) and were performed as previously described [20]. A total of 30 mice were enrolled in the study. One mouse was euthanized before the study endpoint due to tumor ulceration and was excluded from the final analysis. The remaining 29 mice were randomized into treatment groups: Mock/Sham (n = 7), abemaciclib (n = 5), abemaciclib + BUB1i (n = 5), abemaciclib + RT (n = 5), and abemaciclib + BUB1i + RT (n = 7). Mice received abemaciclib (25 mg/kg, orally), BUB1 inhibitor (25 mg/kg, oral gavage), and/or fractionated radiotherapy (5 Gy × 3, conformal irradiation using Small Animal Radiation Research Platform, SARRP, Xstrahl Life Sciences, Surrey, UK) Tumor volumes were measured throughout the study, and growth trajectories were compared across Mock/Sham, abemaciclib alone, abemaciclib + BUB1i, abemaciclib + RT, and abemaciclib + BUB1i + RT treatment groups.

### 2.10. DNA Damage Reporter Assay

Cells carrying the bioluminescent repair reporter were generated previously [20]. Cells were treated as indicated, and secreted *Gaussia* luciferase (Gluc, HR activity) and *Vargula* luciferase (VLuc, NHEJ activity) were measured from the conditioned media collected at the defined time points post-treatment. Luciferase signals were measured with coelenterazine or vargulin substrates, and GLuc/VLuc activities were normalized to the corresponding vehicle control/sham and expressed as relative HR and NHEJ repair activity.

### 2.11. gH2AX, Rad51, RPA and BrdU Foci Assays

DNA damage and repair were evaluated using multiple foci-based assays, including γH2AX, Rad51, RPA, and BrdU as described previously [18,20,49,50]. Cells received 4 Gy irradiation alone or with BUB1i (1 µM), ribociclib (10 µM), or abemaciclib (1 µM); fixation was performed at 2 h (early) and/or 24 h (late) post-RT. Cells were extracted/permeabilized, fixed in 4% paraformaldehyde, blocked, and stained with gH2AX (05-636-25UG, 1:2000, Sigma-Aldrich, St. Louis, MO, USA), Rad51 (65653S, 1:300, Cell Signaling Technology, CST, Danvers, MA, USA), RPA (2208S, 1:400, CST), BrdU (347580, 1:200, BD Biosciences, Franklin Lakes, NJ, USA), and secondary antibodies (A-11004 and A-11011, Thermo Fisher Scientific and 4417S, CST) as described [49,50] with slight modifications. Coverslips were mounted using ProLong Gold Antifade Mountant with DAPI (# P36941, Thermo Fisher Scientific, Waltham, MA, USA). For end-resection readouts, cells were co-stained for RPA and BrdU. All experiments were independently repeated ≥3 times. For each staining, immunofluorescence images were acquired under identical microscope settings. Foci-positive nuclei were defined as >10 foci/nucleus. More than 50 cells (1–2 coverslips) were scored for each condition in all experiments.

Merged images were imported in ImageJ version 1.54f (National Institutes of Health, Bethesda, MD, USA), calibrated using Analyze>Set Scale, converted to composite format, and split into individual channels. The BrdU and RPA channels were merged, background artifacts were removed, and images were saved as JPEGs. Each image was then converted to 8-bit, and a consistent threshold was applied for all. Area-related measurements were selected under Analyze>Set Measurements. Particle analysis was conducted using Analyze>Analyze Particles with options to display and clear results, generate summaries, exclude particles on edges, and show outlines. Output was exported to Excel, and outlined images were saved. To characterize foci size distribution, particles identified by ImageJ were sorted into three size-defined subgroups: 0–0.1 µm, 0.1–0.2 µm, and >0.2 µm. The number of particles within each size category was recorded for every sample and compiled in Excel. A frequency plot was generated using GraphPad Prism to visualize the relative abundance of small, intermediate, and large foci.

Micronucleus formation was assessed using the same DAPI-stained fluorescence microscopy images acquired for the BrdU-RPA foci analyses. Micronuclei were identified as discrete DAPI-positive nuclear bodies separated from the primary nucleus and scored according to established morphological criteria [51].

### 2.12. Comet Assay

DNA damage was evaluated using comet assays adapted from established protocols [52,53,54]. The comet assay is a single-cell gel electrophoresis technique that detects DNA damage under neutral or alkaline conditions, producing a characteristic “comet” consisting of an intact head and a damaged tail [54,55,56]. Neutral comet assays were used to assess DNA double-strand breaks, whereas alkaline comet assays were used to detect total DNA strand breaks and alkali-labile sites. Enzyme-modified alkaline comet assays utilizing formamidopyrimidine DNA glycosylase (FPG; #M0240S, New England Biolabs, (NEB), Ipswich, MA, USA) and Endonuclease III (Nth; #M0268S, New England Biolabs, (NEB), Ipswich, MA, USA) were performed to detect oxidized purine and oxidized pyrimidine lesions, respectively. DNA damage was assessed using the olive tail moment, a sensitive metric defined as the product of the percentage of DNA in the tail and the distance between the head and tail centers of mass [57]. Frosted slides (# EM000W3, Cancer Diagnostics Inc., Durham, NC, USA) were precoated with 1% normal-melting agarose (NMA). Following 24 h treatment, cells were harvested, washed in cold PBS, and resuspended at 1 × 10^6^ cells/mL. A 50 µL aliquot of the suspension was mixed with 500 µL 0.7% low-melting agarose (LMA) and 75 µL of this mixture was layered onto NMA-coated slides. After solidification, a second layer of plain LMA was added, and slides were incubated overnight at 4 °C in lysis buffer.

For the neutral comet assay, slides were lysed in buffer (pH 10) containing 2.5 M NaCl, 100 mM EDTA, 10 mM Tris-HCl, 1% N-lauroylsarcosine, and 0.5% Triton X-100 (added immediately before use). DNA was separated using pre-chilled neutral electrophoresis buffer (300 mM sodium acetate and 100 mM Tris-HCl, pH 8.3). For the alkaline comet assay, slides were lysed in buffer (pH 10) containing 2.5 M NaCl, 100 mM EDTA, and 10 mM Tris-HCl, supplemented immediately before use with 1% Triton X-100 and 10% DMSO. Following lysis, slides were incubated in alkaline electrophoresis buffer (300 mM NaOH and 1 mM EDTA, pH > 13) to allow DNA unwinding prior to electrophoresis (30 min at 4 °C in the dark).

To assess oxidative DNA base damage, the alkaline comet assay was modified by incubating slides with either formamidopyrimidine DNA glycosylase (FPG) or endonuclease III (Nth) after the lysis step. FPG recognizes oxidized purines, whereas Nth recognizes oxidized pyrimidines. These enzymes convert oxidized base lesions into detectable DNA strand breaks [54,55,58,59]. Following lysis, slides were washed three times for 5 min each in 1× FPG reaction buffer. Slides were then incubated with either FPG or Nth enzyme solution (50 μL/gel) in a humidified chamber at 37 °C for 40 min. After enzyme treatment, slides were processed as described for the standard alkaline comet assay.

After electrophoresis, slides were precipitated, dehydrated in 75% ethanol, and stored protected from light. For imaging, slides were rehydrated, stained with ethidium bromide (2 µg/mL), washed, and coverslipped. Comets were visualized by fluorescence microscopy, and ≥50 nuclei per slide were analyzed. DNA damage was quantified using the median olive tail moment to account for non-normal data distributions.

### 2.13. Cell-Cycle Analysis

Cell-cycle assay was performed as described previously [60] with minor modifications. SUM159 cells were seeded in 60 mm dishes and treated for 72 h. Cells were washed with PBS, harvested by trypsinization, collected in culture medium, and centrifuged. The cell pellets were washed twice with PBS and fixed in 70% cold ethanol for 1 h at 4 °C. Following fixation, cells were centrifuged, washed with PBS to remove residual ethanol, and stained in the dark for 30 min with a solution containing 50 μg/mL propidium iodide (PI) and 100 μg/mL RNase A in PBS. Samples were kept on ice and analyzed by flow cytometry. A total of 50,000 events were acquired per sample using a BD LSRFortessa flow cytometer (BD Biosciences) with BD FACSDiva™ v8.0.1 acquisition software. Cell-cycle distribution was determined based on DNA content using ModFit LT™ v3.2 software.

### 2.14. qRT-PCR

Quantitative reverse-transcriptase polymerase chain reaction (qRT-PCR) was carried out as described previously. Primer sequences, reaction conditions and statistical analyses have been described [19].

### 2.15. Immunoblotting

Total protein lysates were prepared in RIPA buffer supplemented with protease and phosphatase inhibitors and processed as described previously [20]. For mechanistic assays, Rb+ SUM159 cells were pre-treated with CDK4/6 inhibitors ribociclib (10 µM) or abemaciclib (1 µM) 24 h before BUB1i (1 µM). ATR inhibitor (BAY1895344 at 150 nM) was used as a positive control. Cells were irradiated an hour later with 4Gy and total cell lysates were prepared in RIPA buffer 2 h and 24 h post-RT. Total proteins were quantified using BCA method and resolved on 4–12% Bis-Tris SDS-PAGE gels.

Additionally, SUM159 cells were transfected with BLM (L2-007287-01-0005, ON-TARGETplus 2.0 Human BLM siRNA, SMARTpool, 5 nmol, (Dharmacon, Horizon Discovery, Lafayette, CO, USA) or EXO1 (#L2-013120-01-0005, ON-TARGETplus 2.0 Human EXO1 siRNA, SMARTpool, 5 nmol, (Dharmacon, Horizon Discovery, Lafayette, CO, USA)) targeting siRNA (80 nM) using Lipofectamine™ RNAiMAX Transfection Reagent (#13778075, Thermo Fisher scientific, Waltham, MA, USA). Following 48 h of transfection, cells were treated with abemaciclib 24 h before BUB1i and ATRi, irradiated an hour later, and lysates were prepared 2 h and 24 h post-RT and run on gels as described above. Knockdown efficiency was confirmed by Western blotting of BLM protein expression.

### 2.16. Statistical Analysis

Response data were analyzed using GraphPad Prism v10. Dose–response curves were generated by plotting normalized cell viability against the logarithm of drug concentration and fitting the data using nonlinear regression (log[inhibitor] vs. normalized response, variable slope). The half-maximal inhibitory concentration (IC_50_) for each treatment was calculated from the fitted dose–response curves. Drug interactions were quantified with the Chow-Talay Combinatory Index (CI), defined as CI = (D)_1_/(Dχ)_1_ + (D)_2_/(Dχ)_2_, where (D)_1_ and (D)_2_ are the doses of drug 1 and drug 2 that, when combined, produce a specific effect, χ%. And (Dχ)_1_ and (Dχ)_2_ are the doses of drug 1 and drug 2 that individually achieve the same effect (χ%), respectively [19,61,62]. CI values were calculated in Microsoft Excel using the Chou–Talalay equation. A C.I. value < 1, =1, and >1 indicated synergism, additivity, and antagonism, respectively. Statistical significance between two groups was determined using an unpaired *t*-test, and significance among multiple groups was assessed using ordinary one-way ANOVA followed by Dunnett’s multiple comparisons test. *p*-values were defined as * = 0.05, ** = 0.01, *** = 0.001, **** = 0.0001. Data was graphed in GraphPad Prism v10 and reported as mean ± SD or SEM.

Time to tumor doubling was analyzed using Kaplan–Meier methods. Tumor doubling time was calculated from the date of tumor measurement to the date when the tumor volume doubled and were reported as days. The Kaplan–Meier curves of tumor doubling time were compared between different treatment groups using log-rank tests, and the median time and the corresponding 95% CI to tumor doubling were reported. IBM SPSS Statistics for Windows (v29) were used for this analysis.

An overview of the experiments with drug treatment timing, radiation schedule, and experimental endpoints is provided in Table S2.

## 3. Results

### 3.1. Correlation and Toxicity Analysis of BUB1 and CDK4/6 Inhibition

To investigate whether BUB1 expression is associated with the CDK4/6 signaling axis, we performed correlation analyses using cBioPortal and examined mRNA expression levels between BUB1 and CDK4/6, along with downstream genes. BUB1 mRNA expression was found to be positively correlated with CDK4, CDK6, E2F1, E2F2, E2F3, E2F7, and CDK2 and negatively correlated with RB1 and CCND1 in the METABRIC dataset (Figure S1A–I). Protein expressions of BUB1, CDK4, CDK6, and RB1 were evaluated in SUM159, BT549, MDA-MB-231, HCC1937, and T47D breast cancer cells as well as in MCF10A breast epithelial cells by Western blotting (Figure S1J). These cell lines expressed varying BUB1, CDK4, CDK6 or RB1 proteins and there was no clear correlation between BUB1 and CDK4, CDK6 or RB1 protein levels.

**Figure 1:**
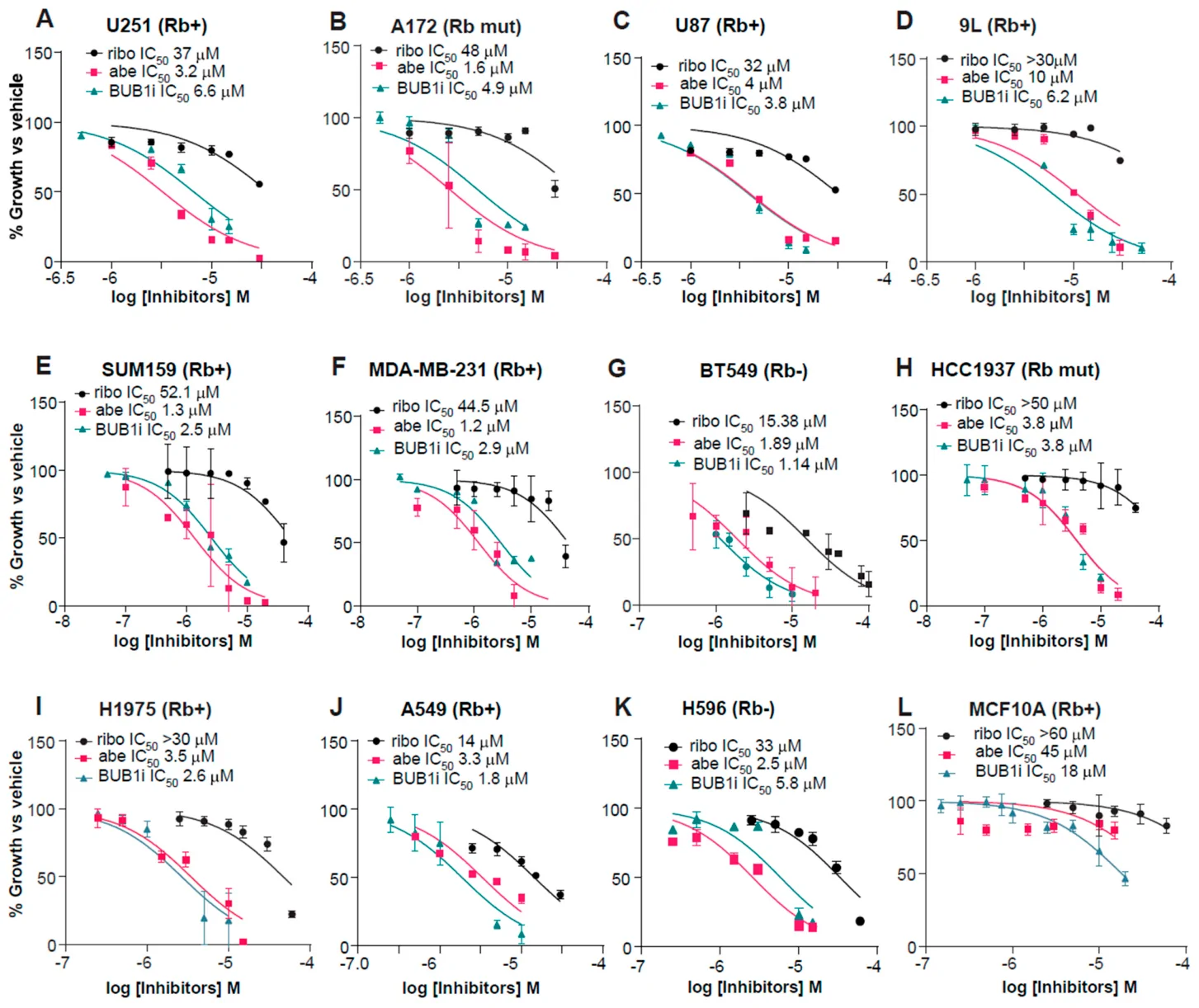
Broad anti-proliferative activity of BUB1i and CDK4/6 inhibitors in glioma, TNBC and lung cancer cell lines. (**A**–**L**) Dose–response growth curves comparing BUB1i, ribociclib (Ribo), and abemaciclib (Abema) in breast (HCC1937, BT549, MDA-MB-231, SUM159), glioma (U251, A172, U87, 9L), lung (H1975, A549, H596) cancer and normal epithelial (MCF10A) cell lines. IC_50_ values are indicated on plots for each agent in each cell line. Experiments were performed in biological triplicates and independently repeated at least three times. Error bars indicate standard deviation (SD) of the mean. Data from representative experiments are shown. Rb+: Rb-positive; Rb-: Rb-null; Rb mut: Rb-mutated.

GBM, LC and TNBC cell lines displayed varying toxicities to a single-agent BUB1 inhibitor, BAY1816032 and CDK4/6 inhibitors, ribociclib and abemaciclib (Table 2). BAY1816032, ribociclib and abemaciclib dose-dependently suppressed cell proliferation of GBM, TNBC, and LC cell lines. The IC_50_ of BUB1i ranged from 3.8 to 6.6 μM in GBM cell lines (Figure 1A–D), 1.14 to 3.8 μM in TNBC (Figure 1E–H) and 1.8 to 5.8 μM in LC cell lines (Figure 1I–K). The IC_50_ was markedly higher at 18 μM, indicating lower toxicity in MCF10A breast epithelial cells (Figure 1L). The IC_50_ of ribociclib ranged from 32 to 48 μM in GBM, 15.38 to 52.1 μM in TNBC, 14 to 33 μM in LC cell lines and >60 μM in MCF10A cells. The IC_50_ of abemaciclib ranged from 1.6 to 10 μM in GBM, 1.2 to 3.8 μM in TNBC, 2.5 to 3.5 μM in LC, and 45 μM in MCF10A cells.

### 3.2. Sequence of BUB1 and CDK4/6 Inhibition Determine Sensitivity to Combination Treatment

**Figure 2:**
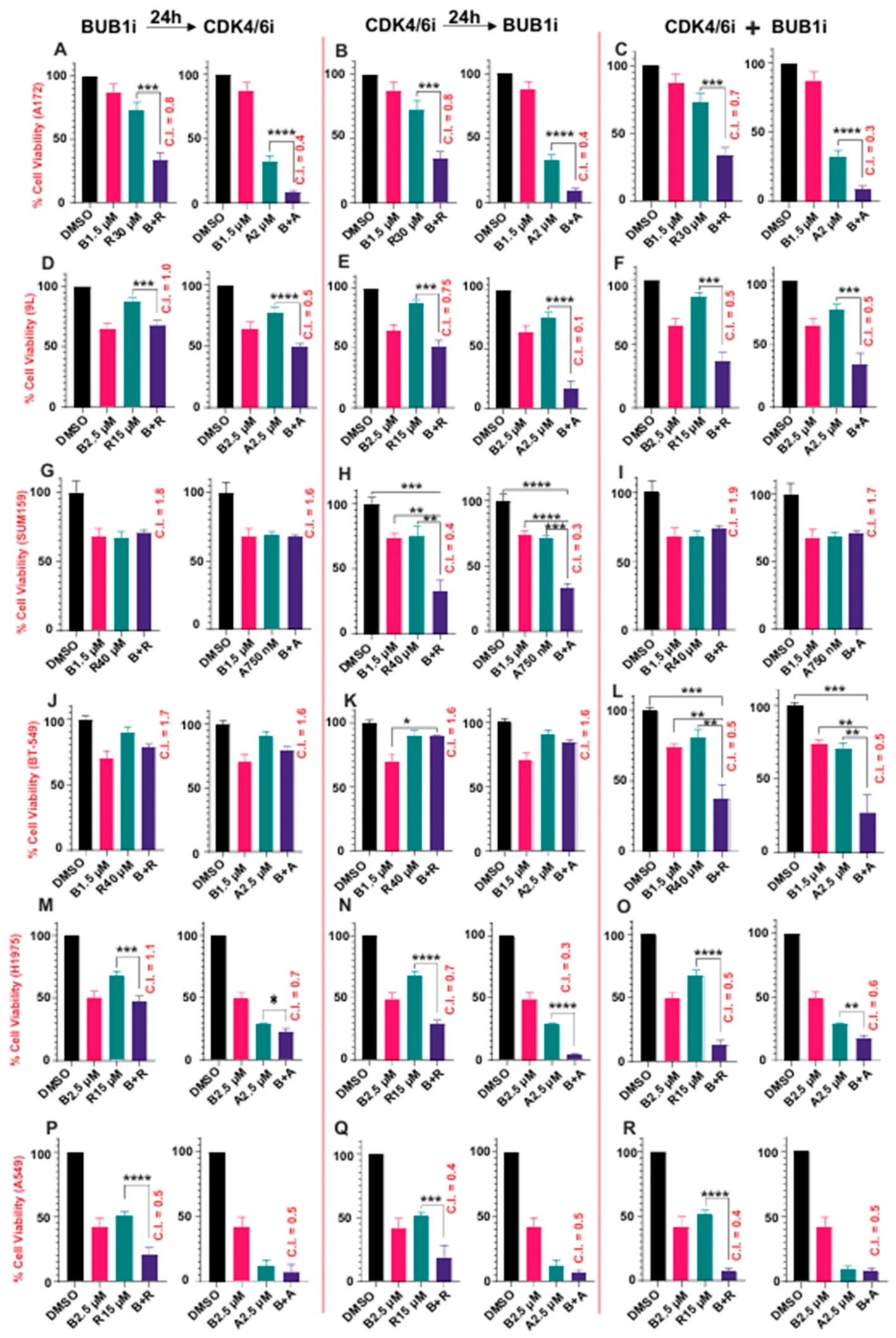
BUB1i with CDK4/6i synergistically reduces viability in a sequence-dependent manner. (**A**–**R**) Short-term viability under DMSO, BUB1i (B), ribociclib (R) or abemaciclib (A) and their combinations in glioma (A172, 9L), breast (BT549, SUM159), and lung (A549, H1975) cancer cell lines. Combination indices (C.I. < 1 denotes synergy, C.I. = 1 denotes additivity, C.I. > 1 indicates antagonism). Error bars indicate SEM/SD of mean. *p*-values: * = 0.05, ** = 0.01, *** = 0.001, **** = 0.0001. Experiments were performed in biological triplicates and independently repeated at least three times. Data from one representative experiment is shown.

Different drug-treatment strategies were explored in these solid cancer cell lines including sequential and concurrent treatments (Figure 2). Single-agent treatments were used as internal controls. In human GBM cell line A172, pretreatment with BUB1i before CDK4/6i, pretreatment with CDK4/6i before BUB1i, and concurrent BUB1i and CDK4/6i treatment all resulted in the same effect (synergistic, CI < 1, Figure 2A–C). In rat gliosarcoma cell line 9L, pretreatment with BUB1i 24 h prior to CDK4/6i resulted in additivity with ribociclib (CI = 1, Figure 2D) and synergism with abemaciclib (CI = 0.5, Figure 2D, right panel). Conversely, pretreatment with ribociclib or abemaciclib followed by BUB1i (Figure 2E) or concurrent treatments (Figure 2F) were synergistic. In human GBM cell line U251 and U87, concurrent BUB1i and CDK4/6i treatment resulted in synergism (Figure S2A,B).

In TNBC cell lines, cytotoxicity with sequential and concurrent BUB1i and CDK4/6i treatments varied based on presence of Rb. In Rb WT (SUM159 and MDA-MB-231) and the Rb mutant (HCC1937) cells, BUB1i pretreatment (Figure 2G and Figure S3A,D) or concurrent treatments were antagonistic (Figure 2I and Figure S3C,F) while sequential treatment with CDK4/6i administered 24 h before BUB1i was synergistic (Figure 2H and Figure S3B,E). Rb-null BT-549 cells exhibited antagonism with sequential treatment (Figure 2J,K) while demonstrating synergism with concurrent treatments (Figure 2L).

In the H1975 LC cell line, pretreatment with BUB1i followed by ribociclib resulted in a nearly additive effect (CI = 1.1, Figure 2M), while pretreatment with BUB1i followed by abemaciclib showed synergism (CI = 0.7, Figure 2M). Both pretreatment with CDK4/6i followed by BUB1i and concurrent treatment resulted in synergistic effects (Figure 2N,O). In the A549 LC cell line, all treatment strategies produced a synergistic effect (Figure 2P–R). In H596 cells, BUB1i pretreatment followed by CDK4/6i was additive or antagonistic (Figure S3G), CDK4/6i pretreatment followed by BUB1i was synergistic (Figure S3H), while concurrent treatment was synergistic or nearly additive (Figure S3I).

### 3.3. Concurrent BUB1 and CDK4/6 Inhibition Enhance Radiation Sensitization in Solid Cancer Cell Lines

**Figure 3:**
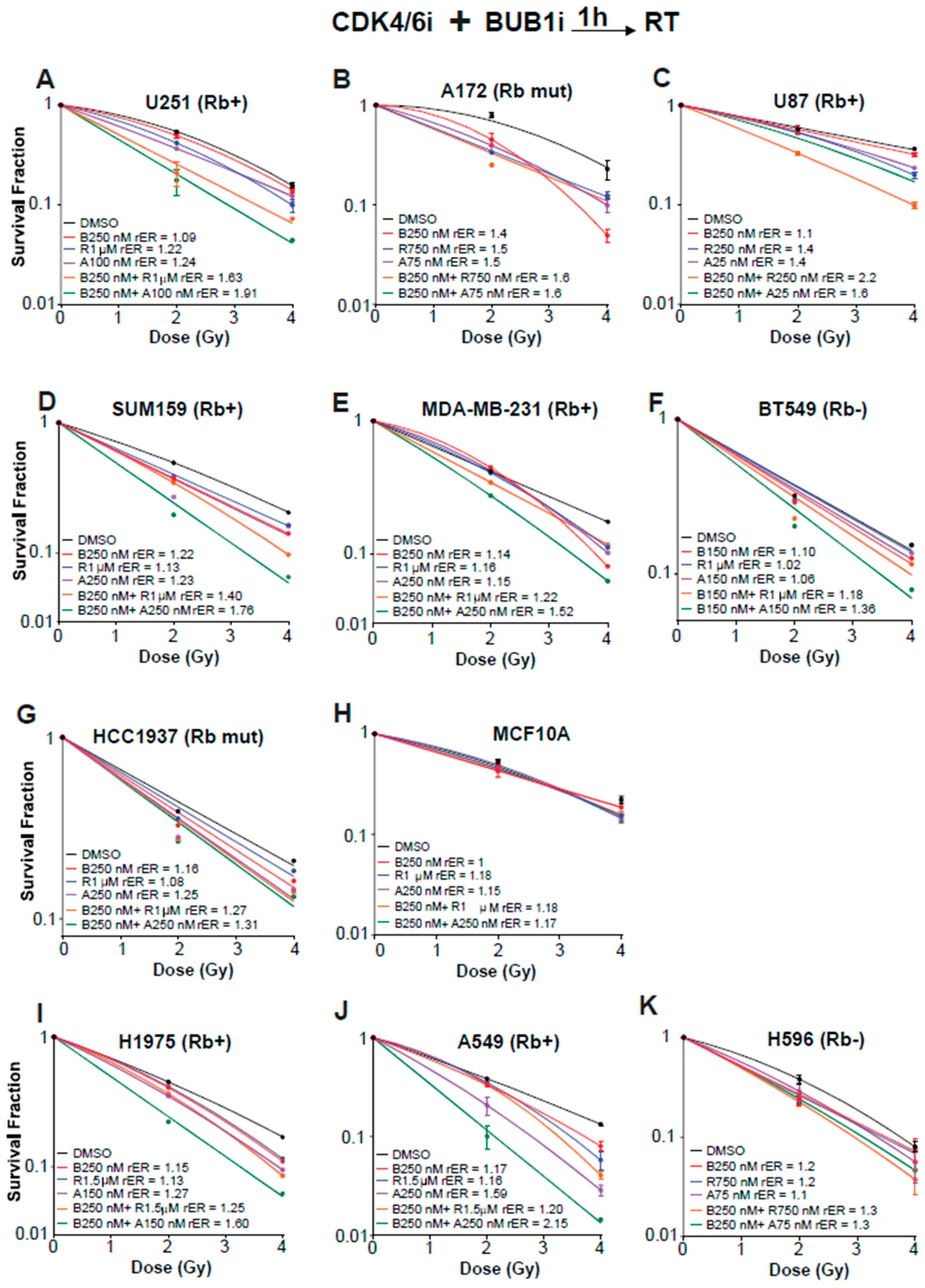
Concurrent BUB1i increases radiosensitization by CDK4/6i in glioma, TNBC and NSCLC cell lines. (**A**–**G**,**I**–**K**) Clonogenic survival after RT with concurrent BUB1i “B”, ribociclib “R”, or abemaciclib “A” in glioma (U251, A172, U87), breast (HCC1937, BT549, MDA-MB-231, SUM159), and lung (H1975, A549, H596) cancer cells. DMSO alone and single drugs were used as controls. Radiation enhancement ratio (rER) was estimated; rER ≥ 1.3 indicates strong enhancement. (**H**) Non-transformed MCF10A was included as a control cell line. All experiments were performed in biological triplicates and independently repeated at least three times; data from one representative experiment is shown.

The effect of combined inhibition of BUB1 and CDK4/6 on radiation sensitization was evaluated across various cancer cell lines using clonogenic assay. Single-agent treatments served as internal controls. Cells were irradiated 1 h after drug treatment. Concurrent BUB1i with ribociclib or abemaciclib increased radiation sensitization (increases in rER compared to a single agent) in Rb+ or Rb- cell lines including glioma (U251, U87), TNBC (SUM159, MDA-MB-231, BT-549), and lung cancer (H1975, A549, H596, Figure 3A–G,I–K). There was insignificant difference in rER between single drugs and combinations in Rb mut glioma (A172, Figure 3B), TNBC (HCC1937, Figure 3G) or normal epithelial MCF10A cells (Figure 3H). PE data at 2 Gy is shown in Figure S4.

### 3.4. Schedule-Dependent Radiosensitization by BUB1i and CDK4/6i Differs by Rb Status

**Figure 4:**
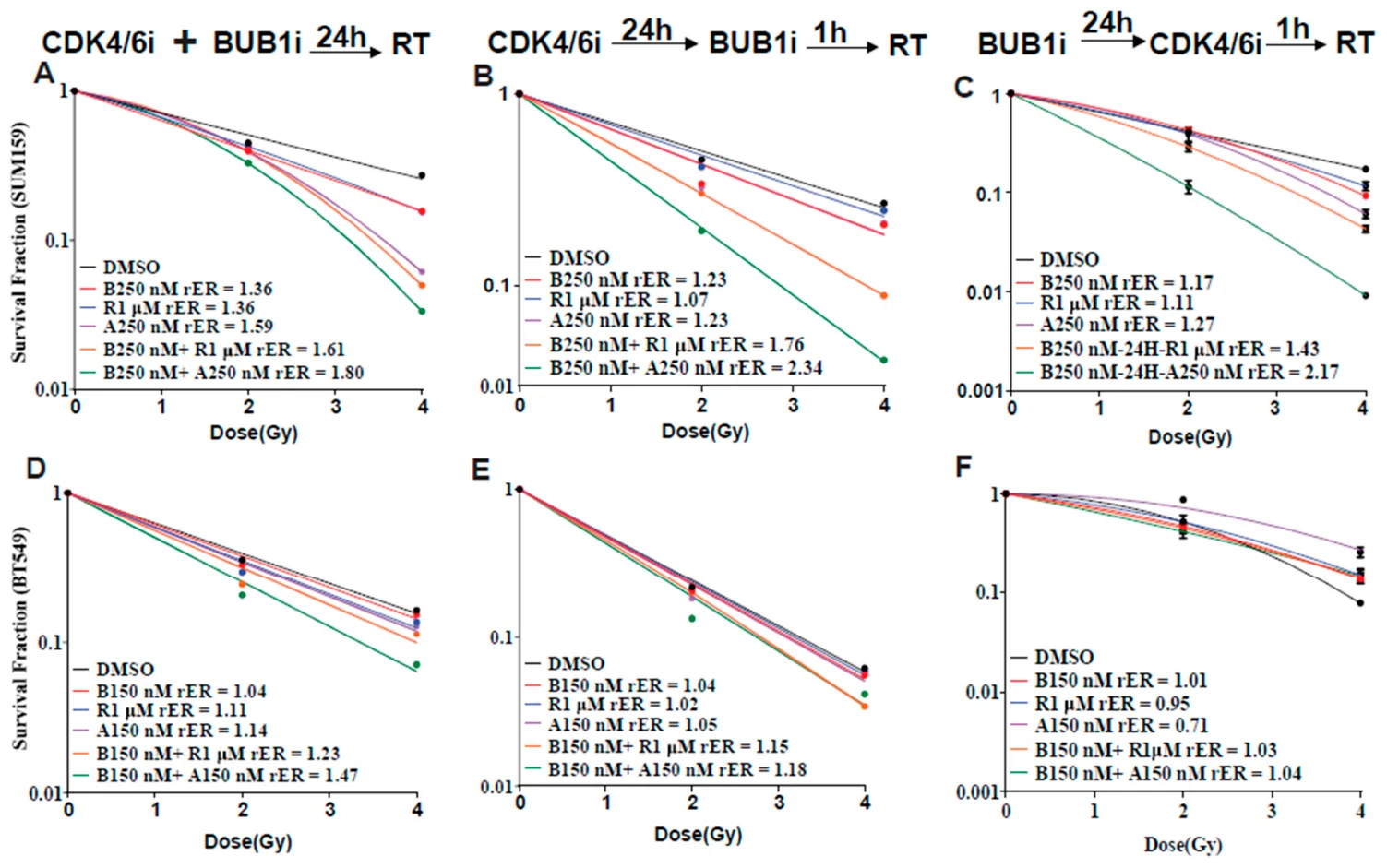
Rb status influences the optimal sequencing of BUB1 and CDK4/6 inhibition for radiosensitization. (**A**–**C**) Clonogenic survival of SUM159 cells (Rb-positive) comparing concurrent and sequential schedules of BUB1i with ribociclib or abemaciclib relative to RT. (**D**–**F**) Clonogenic survival of BT549 cells (Rb null) under the same treatment schedules. Labels above each panel indicate the timing of drug administration relative to RT, and radiation enhancement ratios (rERs) are shown within each plot. DMSO alone and single drugs were used as controls. All experiments were performed in biological triplicates and independently repeated at least three times.

In Rb-proficient SUM159 cells, single-agent BUB1i or CDK4/6i produced modest radiosensitization (rER ~1.06–1.59). Concurrent 24 h cotreatment enhanced radiosensitivity (rER 1.61 with ribociclib, 1.80 with abemaciclib, Figure 4A). Sequential regimens further increased effect size when CDK4/6i was added 24 h before BUB1i (rER 1.76 ribociclib and 2.34 abemaciclib, Figure 4B) or when BUB1i added 24 h before CDK4/6i (rER 1.40 ribociclib and 2.17 abemaciclib, Figure 4C). Survival curves showed the greatest left shift with abemaciclib-containing sequences.

In Rb-deficient BT549 cells, CDK4/6i and BUB1 concurrent treatments increased rER (Figure 4D) while both sequential schedules did not significantly alter radiation response where rER values remained ~1.0–1.15, with survival curves overlapping vehicle controls (Figure 4E,F). PE data at 2 Gy shown in Figure S4.

### 3.5. Genetic Loss of BUB1 Increases Radiosensitization to CDK4/6 Inhibition Across Multiple Cancer Models

To determine whether genetic depletion of BUB1 enhances CDK4/6i mediated radiosensitization, clonogenic survival assays were performed in BUB1 knockout derivatives of SUM159, MDA-MB-231, H1975, and A549 cells. Across all four BUB1-deficient models, CDK4/ inhibition increased sensitivity to radiation, reflected by left-shifted survival curves and elevated radiation enhancement ratios (rERs). In SUM159 BUB1-KO cells (Figure S5A), abemaciclib and ribociclib increased rER values to 1.55 and 1.77, respectively, over parental cells. Similarly, in MDA-MB-231 BUB1-KO cells (Figure S5B), rER values increased to 1.63 with abemaciclib and 1.44 with ribociclib. Lung cancer models demonstrated comparable effects wherein H1975 BUB1-KO cells exhibited rER values of 2.4 (abemaciclib) and 1.81 (ribociclib) (Figure S5C), while A549 BUB1-KO cells showed rER values of 1.86 and 1.52, respectively, over parental cells (Figure S5D).

### 3.6. BUB1 Inhibition Enhances the Antitumor Activity of Abemaciclib and Radiotherapy In Vivo

**Figure 5:**
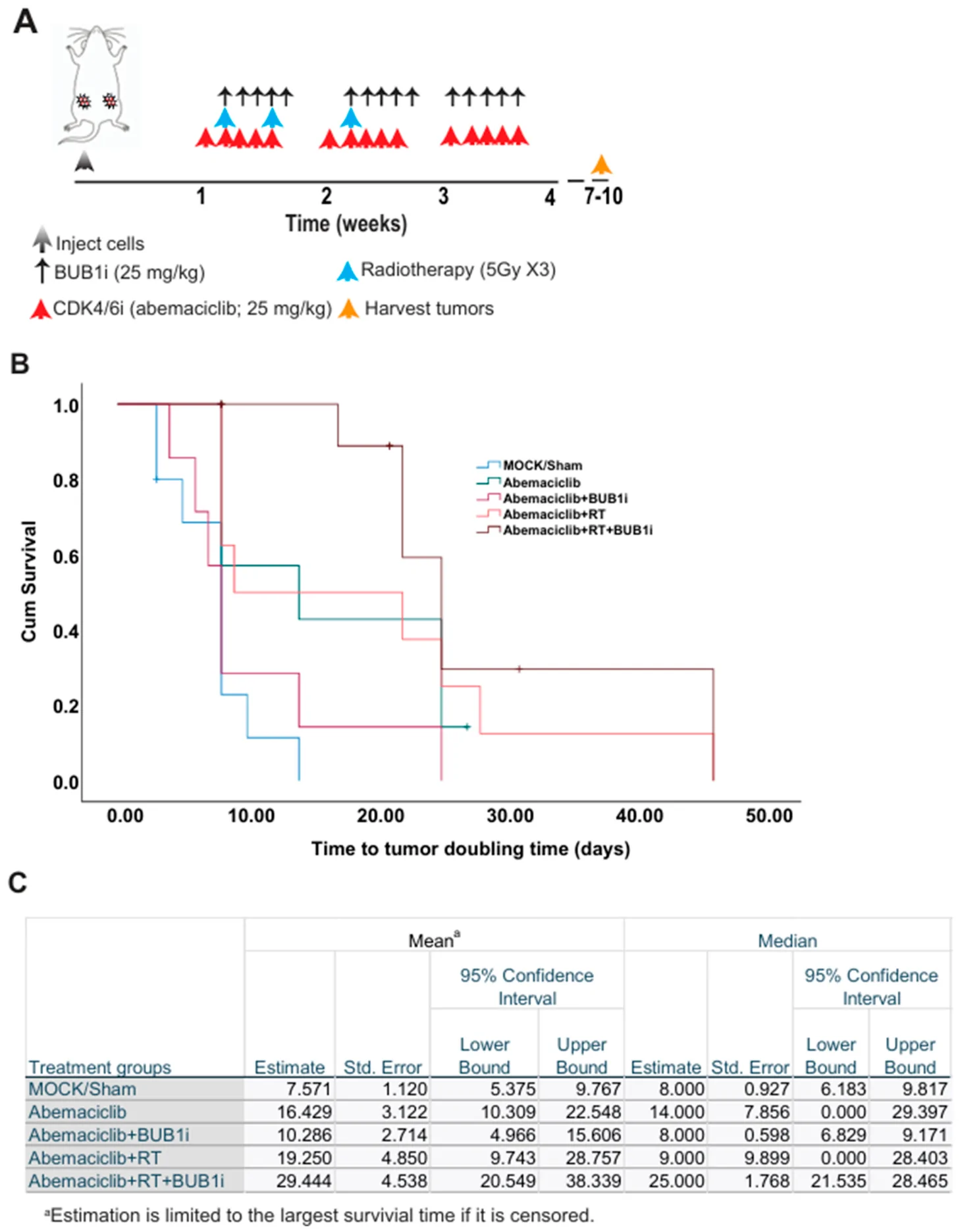
BUB1 inhibition enhances abemaciclib-mediated radiosensitization in vivo under minimally active drug-dosing conditions. (**A**) Treatment schema for bilateral SUM159 mammary fat pad xenografts established in the fourth mammary pad. Drug doses were selected to minimize antitumor effects from single-agent or dual drug treatment alone, thereby allowing assessment of radiosensitization by the triple combination. Abemaciclib and BUB1i were each administered orally at 25 mg/kg, 5 days per week for 3 weeks. Tumors received conformal radiation using SARRP at 5 Gy per fraction for 3 fractions. Five to seven mice were included per treatment group. (**B**) Kaplan–Meier analysis of time to tumor doubling under Mock/Sham, abemaciclib, abemaciclib + BUB1i, abemaciclib + RT, and abemaciclib + BUB1i + RT. (**C**) Mean and median time to tumor doubling for each treatment group. Under these minimally active dosing conditions, the triple combination produced the greatest delay in tumor growth relative to single-agent and dual treatment groups. Animal studies were performed under approved institutional animal care protocols.

To evaluate whether BUB1 inhibition augments CDK4/6 blockade and radiotherapy in vivo, bilateral mammary fat pad tumors were established and treated following the schema (Figure 5A). Mice received abemaciclib (25 mg/kg), BUB1i (25 mg/kg), and/or fractionated radiotherapy (5 Gy × 3), with 5–7 animals per group. Tumor growth was monitored until study endpoint under institutional ethical approvals. These drug concentrations were chosen such that either drug, when administered with radiation, would have minimal to no effect on tumor growth rate. BUB1i alone and BUB1i + RT groups were omitted to minimize the number of mice used for these studies.

Compared to mock/sham treatment abemaciclib, abema + BUB1i, and abema + RT increased mean tumor doubling time (Figure 5B,C) while the median tumor doubling time remained largely unaffected. Notably, the triple combination treatment (Abema + BUB1i + RT) led to highest increase in tumor doubling time from ~8 days (mock/sham) to 29 days (mean) or 25 days (median) in triple combination (Figure 5B,C). Animal body weight remained stable throughout the study (Figure S6). No significant differences were observed between treatment and control groups, and all fluctuations were within ±20% of initial body weight, demonstrating that the treatment regimen was well tolerated and did not cause overt systemic toxicity.

### 3.7. BUB1 Inhibition Resensitizes CDK4/6 Inhibitor Resistant TNBC Cells to CDK4/6 Blockade and Radiotherapy

To investigate whether BUB1 inhibition can overcome acquired resistance to CDK4/6 inhibitors, ribociclib- and abemaciclib-resistant derivatives of SUM159 and BT549 cells were generated by continuous dose escalation (Figure S7). Resistant lines exhibited substantially elevated IC_50_ values (Figure S7A–D) compared to parental cells (Figure 1E,G), confirming successful establishment of drug-resistant phenotypes. In cell viability assays concurrent treatment with BUB1i significantly reduced tumor cell growth (C.I. < 1), indicating synergistic activity (Figure S7E–H). Statistical analyses demonstrated robust resensitization across resistant conditions (*p* < 0.001 to *** *p* < 0.0001).

Clonogenic survival assays showed that BUB1 inhibition increased the radiation response in CDK4/6i-resistant SUM159 cells (Rb+) under both concurrent and sequential dosing schedules, with higher rER values and left-shifted survival curves (Figure S7I,J). In contrast, CDK4/6i-resistant BT549 cells (Rb-) exhibited minimal enhancement of radiosensitivity across the same concurrent and sequential treatments where survival curves remained similar to controls (Figure S7K,L). These findings indicate that BUB1i-mediated radiosensitization is evident in Rb+ (SUM159) but limited in Rb- (BT549) CDK4/6i-resistant models.

Immunoblot analysis shows that BUB1 protein levels increased in both ribociclib-resistant (Ribo^r^) and abemaciclib-resistant (Abe^r^) SUM159 cells compared with parental SUM159 cells (Figure S8), indicating potential association of BUB1 upregulation with acquired CDK4/6 inhibitor resistance. Treatment with BUB1 inhibitor reduces BUB1 protein levels in CDK4/6i-resistant cells to that of the parental SUM159 level, supporting BUB1 as a therapeutically actionable vulnerability in CDK4/6i-resistant settings.

### 3.8. BUB1 and CDK4/6 Inhibition Alter DNA Repair Pathway Activation Following Radiotherapy

To determine how BUB1 and CDK4/6 inhibition influence DNA damage repair, homologous recombination (HR, GLuc) and non-homologous end-joining (NHEJ, VLuc) reporter assays were performed in SUM159 and BT549 cells under radiotherapy with or without the indicated inhibitors (Figure S9A–D). In SUM159 cells, radiotherapy alone modestly increased HR activity while the combination of BUB1i or CDK4/6i with RT further elevated HR repair efficiency (*p* < 0.01–0.001), with the greatest increase observed under dual-inhibitor plus RT conditions (Figure S9A). Conversely, NHEJ activity was reduced with BUB1i + RT and further suppressed with dual-inhibitor combinations (*p* < 0.01–0.001), indicating a shift away from NHEJ repair (Figure S9B). A similar pattern was observed in BT549 cells. HR activity increased across all RT + inhibitor combinations (Figure S9C), whereas NHEJ efficiency significantly reduced with BUB1i, CDK4/6i, and especially with their combination with RT (*p* < 0.0001, Figure S9D). Together, these results indicate that BUB1i and CDK4/6i modulate DNA repair pathway choice following radiotherapy, resulting in increased HR activity and reduced NHEJ reporter activity in both TNBC models evaluated.

### 3.9. Combination Therapy Increases DNA Damage and Unresolved HR-Associated Intermediates at 24 h After RT

**Figure 6:**
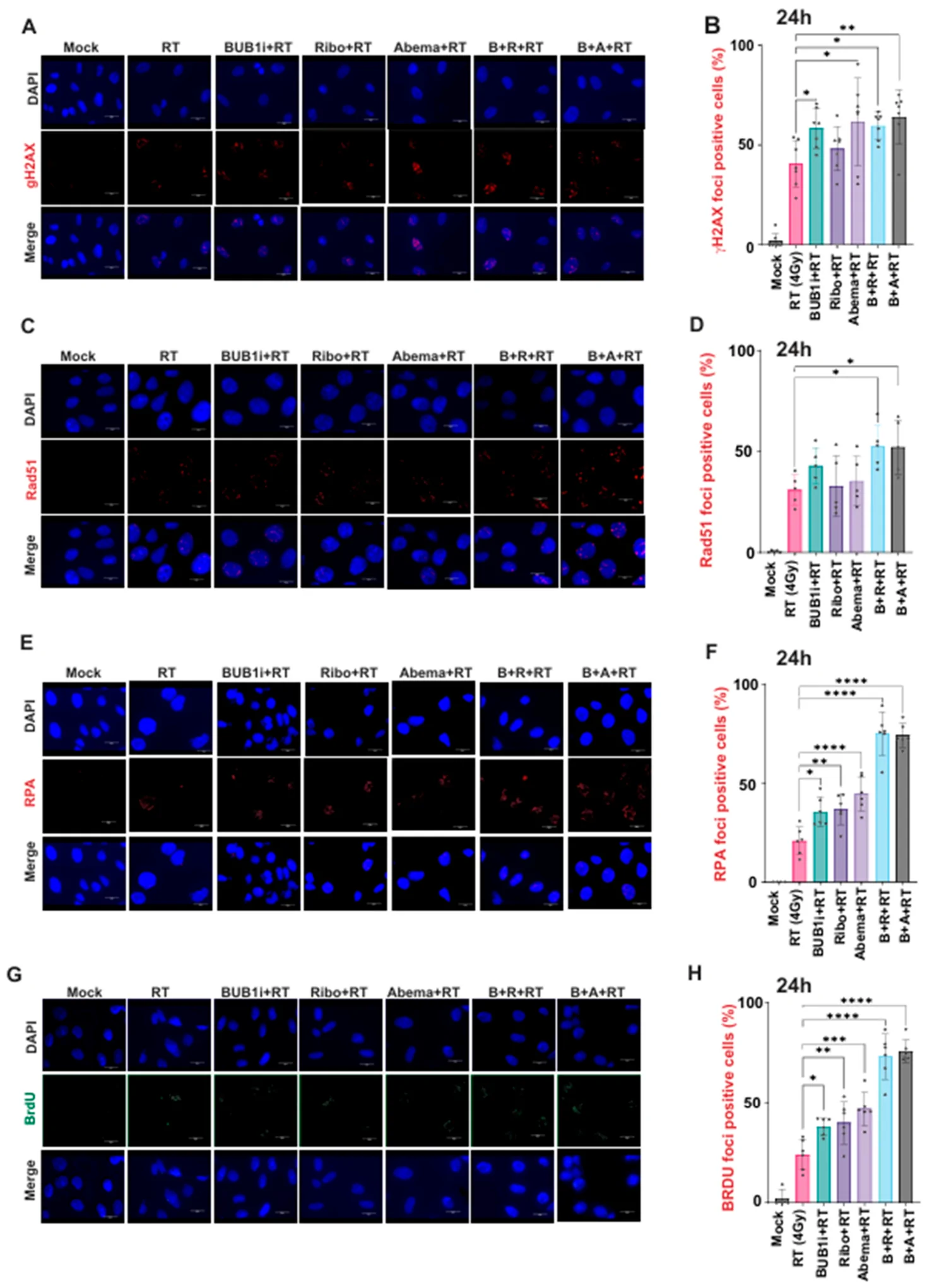
Combination therapy increases persistent DNA damage and HR-associated repair intermediates. (**A**–**D**) gH2AX and RAD51 foci 24 h after RT (4 Gy) ± BUB1i (B, 1 µM), ribociclib (R, 10 µM), abemaciclib (A, 1 µM), B + R+RT, or B + A+RT. Foci-positive SUM159 cells (>10 foci/nucleus) were counted and presented as percent (%) with statistical significance. (**E**–**H**) RPA and BrdU foci-positive cells were counted under the same treatment conditions. At least 50 individual cells were counted for each experiment (1–2 coverslips), and all the experiments were independently repeated at least three times. Data is combined from 3–5 experiments, and each datapoint in the bar graph represents an individual coverslip with 25–50 cells. Scale bars represent 25 µm. gH2AX, RAD51, and RPA staining are shown in the TRITC channel (red), BrdU staining is shown in the FITC channel (green), and nuclei are counterstained with DAPI (blue). Error bars indicate SD of the mean. Statistical significance was determined using ordinary one-way ANOVA with Dunnett’s multiple comparisons test. *p*-values were defined as * = 0.05, ** = 0.01, *** = 0.001, **** = 0.0001.

To define how combined BUB1 and CDK4/6 inhibition alters the DNA damage response after radiotherapy, we quantified gH2AX, RAD51, RPA, and BrdU foci 24 h after treatment in SUM159 cells (Figure 6A–H). Radiotherapy increased gH2AX relative to mock, and addition of either BUB1i or CDK4/6i further increased the fraction of gH2AX-positive cells, with the highest levels observed in the dual-inhibitor plus RT groups (*p* < 0.05 to 0.01, Figure 6A,B). These data indicate increased persistence of DNA damage under combined treatment. RAD51 foci followed a similar pattern and were highest at 24 h under the BUB1i plus CDK4/6i plus RT conditions (*p* < 0.05 to 0.01, Figure 6C,D), indicating increased accumulation or persistence of HR-associated factors.

Markers of DNA end resection also increased at 24 h. Both RPA and BrdU foci were elevated by RT and further increased by drug combinations, with maximal accumulation in the triple-therapy groups (*p* < 0.001 to 0.0001, Figure 6E–H). Importantly, at the early 2 h time point, there were no significant differences among treatment groups in the proportion of cells positive for RPA, BrdU, or RPA-BrdU colocalized foci (Figure S9E–G), indicating that initial entry into resection is not grossly altered shortly after irradiation. Thus, the major effect of combined BUB1 and CDK4/6 inhibition emerges at later time points and is characterized by persistent DNA damage, sustained ssDNA-associated structures, and prolonged HR-associated factor retention. Together, these findings are consistent with impaired resolution of DNA damage repair intermediates and altered HR repair progression.

### 3.10. Comet Assay Demonstrates Enhanced DNA Damage Following Combined BUB1 and CDK4/6 Inhibition with Radiotherapy

The effect of combined BUB1 and CDK4/6 inhibition with radiotherapy on DNA damage was evaluated using comet assays. At 24 h post-RT, treatment with BUB1i or CDK4/6i with radiotherapy increased the olive moment relative to radiotherapy alone, indicating greater residual DNA damage (Figure S10A–E). However, the dual inhibitor with radiotherapy produced the highest olive moment (*p* = **, 0.01 and *p* = ****, 0.0001), suggesting that simultaneous inhibition of BUB1 and CDK4/6 further compromises DNA repair capacity that leads to sustained DSB accumulation. Furthermore, FPG- (Figure S10D) and Nth-modified (Figure S10E) comet assays demonstrated higher olive moment values than standard alkaline comet assays (Figure S10C), revealing the presence of oxidative DNA base lesions in addition to strand breaks [54].

### 3.11. BUB1 and CDK4/6 Co-Inhibition Promotes Persistent Resected DNA Intermediates but Destabilizes Late ATR-Dependent Repair Signaling

**Figure 7:**
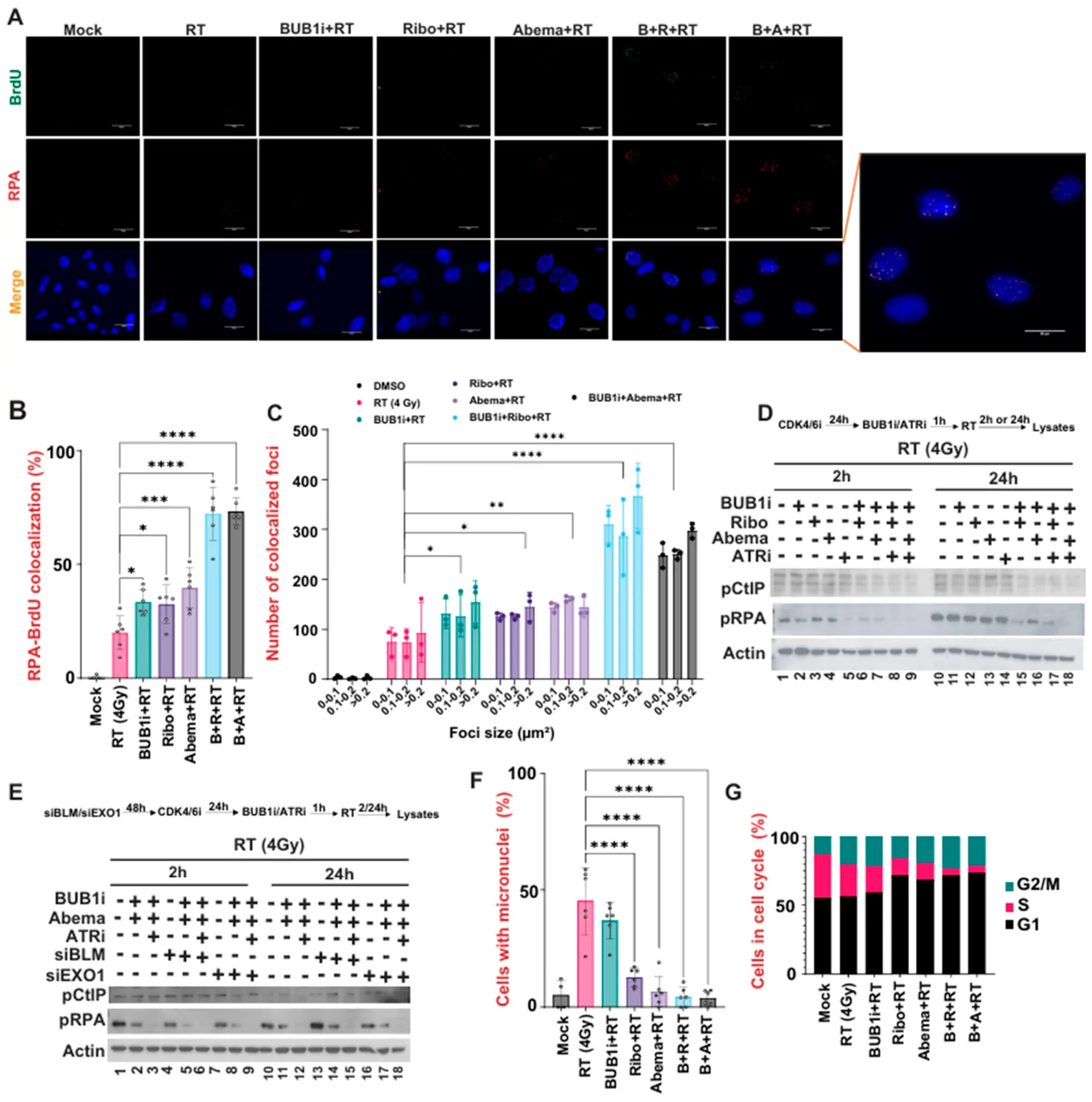
Combined BUB1 and CDK4/6 inhibition with RT increases resection-associated foci and alters late damage signaling. (**A**) Representative RPA and BrdU dual-stain images obtained 24 h post-RT in SUM159 cells. Inset shows RPA-BrdU colocalized foci. Scale bars represent 25 µm. (**B**) Quantification of cells with RPA BrdU colocalization 24 h after treatment. (**C**) Size distribution of RPA-BrdU colocalized foci. (**D**) Immunoblot analysis of DNA damage response markers in SUM159 cells treated sequentially with CDK4/6 inhibitors (ribociclib or abemaciclib, 24 h lead-in) followed by BUB1i and RT (4 Gy). pCtIP S327 and actin served as resection initiation and loading controls. (**E**) Immunoblot analysis of pCtIP and pRPA in SUM159 cells treated with RT in the presence of control siRNA, siBLM, or siEXO1, with or without BUB1i, abemaciclib, or the ATR inhibitor. (**F**) Quantification of micronucleus positive cells 24 h after RT. (**G**) Cell-cycle distribution of SUM159 cells under mock, single-agent, dual-combination, and triple-combination conditions. For foci experiments (**A**–**C**,**F**), at least 50 cells were scored per experiment from 1 to 2 coverslips, and all experiments were independently repeated at least three times. Bar graph data are combined from 3 independent experiments, with each point representing one coverslip containing 25 to 50 cells. RT was delivered at 4 Gy. BUB1i was used at 1 µM, ribociclib at 10 µM, and abemaciclib at 1 µM. BrdU staining is shown in the FITC channel (green), RPA staining is shown in the TRITC channel (red), and nuclei are counterstained with DAPI (blue). Error bars indicate SD. Statistical significance was determined by one-way ANOVA with Dunnett’s multiple comparisons test. * *p* < 0.05, ** *p* < 0.01, *** *p* < 0.001, **** *p* < 0.0001.

To determine whether the late phenotype reflected increased resection or failed processing of resected intermediates, we examined RPA BrdU colocalization together with pRPA32 Ser33 and pCtIP after RT (Figure 7A–E). Dual staining showed that RT increased RPA BrdU colocalization relative to mock, and addition of either BUB1i or CDK4/6i further increased the fraction of dual positive cells. The BUB1i plus CDK4/6i plus RT conditions produced the highest colocalization frequencies (Figure 7A,B), with corresponding increases in the number and size distribution of colocalized foci (Figure 7C). These effects were most evident in the larger foci classes, indicating persistence and expansion of ssDNA containing repair intermediates under maximal treatment pressure. Consistent with these findings, the triple-therapy groups also showed higher numbers of RPA and BrdU foci per nucleus (Figure S9H,I). Thus, these findings indicate an accumulation of resected DNA structures at later time points following RT in the presence of combined BUB1 and CDK4/6 inhibition.

We next examined whether this phenotype was associated with changes in ATR-associated resection signaling in Rb+ SUM159 cells (Figure 7D). At 2 h post-RT, ATR inhibition markedly reduced pRPA compared with RT alone and single-agent BUB1i or CDK4/6i plus RT, confirming that early RPA phosphorylation is ATR-dependent (lane 5 vs. lanes 2 to 4). At 24 h, however, the pattern diverged. Single-agent BUB1i or CDK4/6i with RT maintained relatively high pRPA (lanes 11 to 13), whereas the combined BUB1i plus CDK4/6i plus RT conditions showed reduced pRPA (lanes 15 to 17), despite the marked persistence of RPA-BrdU-positive foci. These findings suggest that combined BUB1 and CDK4/6 inhibition alters the maintenance of ATR-RPA signaling at later time points while resected DNA structures remain detectable. Collectively, the data is consistent with altered processing or resolution of DNA repair intermediates following RT, although the precise underlying mechanism was not directly assessed.

To further test whether BUB1 acts at the resection branchpoint and whether this state depends on canonical long-range resection machinery, we repeated the analysis in control, siBLM, and siEXO1 backgrounds in SUM159 cells (Figure 7E and Figure S12). At 2 h post-RT, dual BUB1i plus abemaciclib reduced pRPA relative to RT alone in control, siBLM, and siEXO1 cells (lane 2 vs. lane 1, lane 5 vs. lane 4, and lane 8 vs. lane 7), while ATR inhibition nearly eliminated pRPA in all three backgrounds (lanes 3, 6, and 9). pCtIP changed little across these conditions, indicating that entry into resection remains intact. The early reduction in pRPA with BLM or EXO1 depletion suggests that the magnitude of the early signal may be influenced by resection extension, while resection initiation appears largely preserved.

A distinct pattern emerged at 24 h post-RT. In control siRNA cells, dual BUB1i plus abemaciclib again reduced pRPA relative to RT alone (lane 11 vs. lane 10), indicating altered maintenance of ATR-RPA signaling at later time points. In contrast, BLM depletion increased pRPA under RT alone (lane 13 vs. lane 10), indicating accumulation of unresolved resected intermediates when BLM-dependent processing is impaired. Even in this setting, dual treatment reduced pRPA relative to siBLM plus RT alone (lane 14 vs. lane 13), indicating that combined BUB1 and CDK4/6 inhibition is associated with reduced pRPA signaling despite the persistence of resection-associated structures. EXO1 depletion lowered late pRPA overall (lane 16 vs. lane 10), and dual treatment caused a further modest decrease (lane 17 vs. lane 16), indicating that EXO1 contributes to maintenance of the late resection signal. Across all conditions, pCtIP largely remained stable. Taken together, these data place BUB1 downstream of resection entry and support a model in which combined BUB1 and CDK4/6 inhibition generates persistent resected intermediates but destabilizes the ability to maintain late ATR-dependent signaling. In this framework, EXO1 supports persistence of the late signal, whereas BLM facilitates processing or resolution of resected DNA intermediates that would otherwise accumulate.

### 3.12. Combined BUB1 and CDK4/6 Inhibition Reduces Micronucleus Formation While Enforcing a CDK4/6-Dominant G1-Arrested State

We next asked whether the persistent DNA damage observed under triple therapy was associated with increased chromosome breakage or mitotic missegregation. Micronuclei were quantified 24 h after treatment (Figure 7F). RT alone markedly increased the percentage of cells with micronuclei compared with mock controls. Single inhibitor combinations produced variable effects, with BUB1i plus RT showing a modest reduction and CDK4/6i plus RT remaining elevated relative to mock. In contrast, the triple-therapy groups (BUB1i + CDK4/6i + RT) showed the lowest micronucleus frequencies and were significantly reduced relative to RT alone and single-agent combinations (**** *p* < 0.0001). These data indicate that the combined treatment does not increase micronucleus formation at 24 h but instead suppresses it, arguing against enhanced mitotic passage with chromosome breakage or missegregation as the dominant outcome at this time point.

To determine whether altered cell-cycle distribution could account for this phenotype, we profiled SUM159 cells across four independent biological experiments (Figure 7G). As expected, CDK4/6 inhibition caused marked G1 accumulation with a corresponding reduction in S-phase entry, serving as an internal positive control for CDK4/6 pathway engagement. When BUB1i was combined with CDK4/6i, the G1-arrested phenotype remained dominant and cell populations stayed strongly G1 enriched, with relatively reduced G2/M fractions similar to CDK4/6i + RT alone. This reproducible pattern indicates that CDK4/6i-driven G1 control supersedes the G2/M checkpoint effects of BUB1 blockade in this setting. Together with the reduced micronucleus phenotype, these findings support a model in which combined treatment traps cells in a repair-defective but mitotically constrained state, thereby favoring persistence of unresolved DNA damage over immediate chromosome segregation errors.

### 3.13. Combined BUB1 and CDK4/6 Inhibition Drives Persistent Resected Intermediates and Failed HR Completion After RT

**Figure 8:**
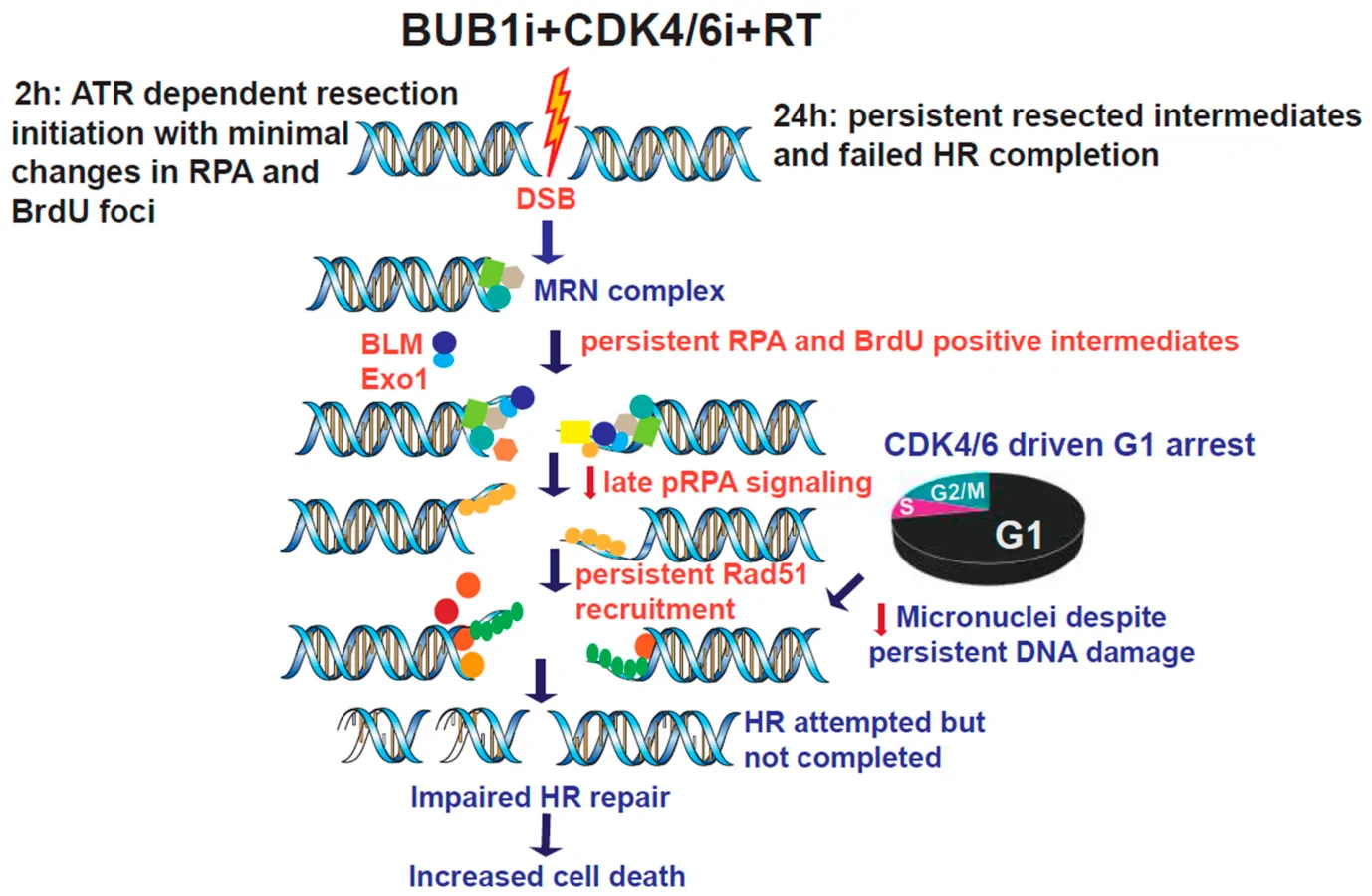
Model of persistent resected intermediates and failed HR completion under combined BUB1 and CDK4/6 inhibition with radiotherapy. Schematic model of the proposed mechanism by which combined BUB1 and CDK4/6 inhibition with radiotherapy disrupts double-strand break repair. At 2 h after irradiation, resection entry is preserved and remains ATR-dependent, with minimal change in RPA and BrdU foci across treatment groups. At 24 h, combined treatment produces persistent DNA damage marked by sustained gH2AX, persistent RAD51 recruitment, increased RPA and BrdU foci, longer olive tail moment, and enhanced RPA-BrdU colocalization with larger foci, consistent with accumulation of persistent resected DNA intermediates rather than repair completion. In parallel, late pRPA signaling is reduced, indicating loss of sustained ATR-dependent repair signaling despite persistence of resected structures. These findings support a model in which HR is engaged but not completed under dual BUB1 and CDK4/6 inhibition. Concurrent CDK4/6i also enforces G1 arrest, limiting mitotic progression and reducing micronucleus formation despite persistent DNA damage. Together, these events result in defective repair completion and increased cell death.

To summarize the mechanistic findings (Figure 8), we propose that combining BUB1 and CDK4/6 inhibition with radiotherapy does not block early entry into DNA end resection but instead alters the fate of repair intermediates over time. At 2 h after RT, resection initiation remains intact and ATR-dependent, with little change in RPA or BrdU foci across treatment groups. By 24 h, however, dual inhibition causes marked accumulation of gH2AX, RAD51, RPA, and BrdU foci, along with increased RPA-BrdU colocalization and larger foci, indicating persistence of resected DNA intermediates and unresolved damage.

Despite this buildup of resection-associated structures, late pRPA signaling is reduced under combined treatment, suggesting loss of sustained ATR-dependent repair signaling rather than repair completion. These findings support a model in which BUB1 and CDK4/6 co-inhibition drives HR engagement but prevents its successful completion, resulting in persistent repair failure. In parallel, CDK4/6i-driven G1 arrest limits mitotic entry and reduces micronucleus formation, placing cells in a repair-defective but mitotically constrained state that ultimately promotes apoptotic cell death (Figure S11).

## 4. Discussion

BUB1 and CDK4/6 have emerged as attractive therapeutic targets in solid tumors because of their important roles in mitosis and cell-cycle regulation [30,63]. Both pathways are frequently overexpressed or hyperactivated in cancers [15,64] and higher expression has been associated with aggressive tumor behavior and worse outcomes in multiple tumor types [24,25,26,27,28,29]. However, the therapeutic landscape is not uniform clinically. CDK4/6 inhibition is established in hormone receptor-positive/HER2-negative breast cancer, whereas its role in TNBC, GBM, and NSCLC remain investigational. Because BUB1 inhibition compromises DSB repair by perturbing NHEJ factor function at breaks [20] and CDK4/6 inhibition induces replication stress with reduction in HR repair programs [18,65], their combination is mechanistically poised to increase DSB repair failure and produce selective cytotoxicity in solid tumors including TNBC. Here, we explored the therapeutic potential of co-targeting BUB1 and CDK4/6 with RT to optimize treatment strategies for solid tumors.

Despite a positive correlation between BUB1 mRNA and CDK4/6 expression in the METABRIC cohort (>2500 tumors), indicative of potential co-regulation, our TNBC cell line panel showed no correlation at the protein level among BUB1, CDK4/6, and RB (Figure S1A–J). This suggests a context-dependent regulation and the need to interrogate pathway function rather than relying solely on mRNA expression correlation. BUB1i (BAY1816032) and CDK4/6i (ribociclib and abemaciclib) were found to suppress the proliferation of GBM, TNBC, and NSCLC cell lines in a dose-dependent manner (Figure 1A–K). In contrast, MCF10A cells required substantially higher doses of BAY1816032, ribociclib, and abemaciclib for growth suppression, supporting a preliminary therapeutic window (Figure 1L).

CDK4/6 inhibitors enforce a G1 arrest [30], limit S-phase entry and proliferation while BUB1 is a core mitotic checkpoint kinase that safeguards chromosome segregation at G2/M [66]. Co-targeting these non-overlapping cell-cycle nodes narrows proliferative “escape routes” and creates complementary pressure on tumor growth. Consistent with this model, our combination studies show schedule- and context-dependent synergy. In GBM models, CDK4/6i pretreatment and concurrent BUB1i + CDK4/6i produced broadly similar synergy (Figure 2A–F and Figure S2). In Rb-intact TNBC, 24 h CDK4/6i pretreatment before BUB1i yielded the strongest synergy (Figure 2G–I and Figure S3A–C), consistent with prior observations that CDK4/6i lead in can enhance combination efficacy in Rb-proficient settings [67]. By contrast, in Rb-null BT549, concurrent dosing performed best (Figure 2J–L) and in NSCLC lines, all three schedules achieved additive-to-synergistic effects (Figure 2M–R and Figure S3G–I). Together, these findings support the pairing of BUB1i with CDK4/6i to exploit G1/S-G2/M complementation and that sequence matters with CDK4/6i lead-in favored in Rb-proficient settings, and concurrency effective when Rb is lost. Importantly, the differential responses observed between Rb-intact and Rb-deficient models suggest that functional Rb signaling may be required for optimal efficacy and support Rb expression as a potential predictive biomarker. Consistent with this, Pesch et al. demonstrated that RB expression predicts sensitivity to CDK4/6 inhibitor-mediated radiosensitization in both ER-positive and triple-negative breast cancer models, whereas RB loss attenuates therapeutic response [18]. Future studies should determine whether Rb-guided patient stratification would be useful in determining the clinical efficacy of combined BUB1 and CDK4/6 inhibition when this combination is evaluated in clinical settings. Notably, broader pharmacology supports schedule aware use of CDK4/6 inhibitors with molecularly targeted or cytotoxic chemotherapy, reinforcing the importance of timing in combination regimens.

Concurrent BUB1i + CDK4/6i at sub-IC_50_ doses consistently increased radiosensitivity across glioma, TNBC, and lung cancer, supporting a combination where BUB1 inhibition weakens NHEJ while CDK4/6 inhibition dampens HR/replication, thus limiting DSB resolution (Figure 3A–G,I–K). MCF10A cells showed minimal change in rER (Figure 3H), again suggesting a potential therapeutic window. Across models, concurrent dosing was effective. Where tested, CDK4/6i lead-in further increased rER in Rb-proficient settings, whereas concurrency was favored in Rb-null models, highlighting G1/S-G2/M complementation (Figure 4) and suggesting that scheduling mattered. Genetic validation further aligned with this mechanism as BUB1 CRISPR KO cells exhibited further loss of clonogenic survival with CDK4/6i ± RT, confirming that dual targeting of NHEJ (BUB1) and HR/replication (CDK4/6) cooperatively sensitizes tumors to radiation (Figure S5). These results are similar to ones observed with ER degraders in CDK4/6i-resistant ER+ models [68]. In vivo, the triple combination of BUB1i + CDK4/6i + RT produced the greatest increase in tumor doubling time, outperforming single-agent and dual-agent regimens and demonstrating the therapeutic advantage of jointly targeting BUB1 and CDK4/6 during radiotherapy (Figure 5). Animal body weight also remained stable (Figure S6), indicating that the triple combination was well tolerated.

CDK4/6i resistant SUM159 and BT549 cells (Figure S7A–D) showed reduced sensitivity to ribociclib/abemaciclib versus parental lines (Figure 1E,G) but BUB1i restored drug response (Figure S7E–H). Notably, BUB1i + CDK4/6i further resensitized Rb-proficient SUM159 to radiation, whereas Rb-null BT549 were not radiosensitized under any schedule (Figure S7I–L), indicating Rb-dependent benefit in the resistant setting. Together, these findings support a strategy in which BUB1 targeting helps overcome CDK4/6i resistance and enhances RT response in Rb-positive tumors, as Rb dependency for radiosensitization in breast cancer has been shown previously. Consistent with this functional reversal, BUB1 protein levels were elevated in ribociclib- and abemaciclib-resistant SUM159 cells, and pharmacologic BUB1 inhibition effectively suppressed this adaptive upregulation, implicating BUB1 as a mechanistically relevant mediator of acquired CDK4/6i resistance (Figure S8).

BLRR demonstrated that combining BUB1i and CDK4/6i with RT modestly increased HR readouts and decreased NHEJ activity relative to RT alone, consistent with a shift toward resection-associated repair under triple therapy (Figure S9A–D). However, this data does not support a simple model of uniform HR suppression. Instead, across models, the triple combination increased gH2AX, RAD51, RPA, BrdU foci and olive tail moment at 24 h, enhanced RPA-BrdU colocalization, and shifted RPA and BrdU foci size toward larger size classes (Figure 7, Figures S9H,I and S10). In addition, increased DNA migration following FPG and Nth digestion indicates the accumulation of oxidative DNA base lesions in response to radiation combined with BUB1 and CDK4/6 inhibition (Figure S10A,D,E). Together, these findings indicate persistent DNA damage and accumulation of resected repair intermediates rather than efficient HR completion.

RPA phosphorylation further supports this model (Figure 7D). At 2 h, ATR inhibition reduced pRPA32 Ser33, confirming ATR-dependent early resection, whereas at 24 h, single-agent BUB1i or CDK4/6i maintained high pRPA but the dual combination reduced pRPA despite persistent RPA-BrdU foci. Consistent with this, siBLM increased and siEXO1 decreased late pRPA, supporting persistent resected intermediates with impaired late ATR-RPA signaling rather than productive HR completion [69,70,71]. Our results are not unexpected as DNAPKcs is known to regulate DNA end resection [72,73], which we earlier identified to phosphorylate and stabilize with BUB1 inhibition [20]. Moreover, CDKs also regulate DNA end resection [74].

Importantly, enhanced radiosensitization was not accompanied by increased micronucleus formation at 24 h (Figure 7F). Instead, persistent DNA damage and unresolved repair intermediates accumulated in a cell-cycle state dominated by CDK4/6i-induced G1 arrest (Figure 7G). This likely limits mitotic progression of damaged cells, thereby suppressing micronucleogenesis (allowing nuclear damage foci to persist) while reducing chromosome segregation errors. Thus, checkpoint enforcement appears to uncouple foci persistence from micronuclear burden and may explain why the combination enhances radiosensitivity without increased mitotic catastrophe.

Mechanistically, these results support a model in which BUB1 inhibition disrupts productive NHEJ progression, while CDK4/6 inhibition limits HR recovery programs, together driving unresolved DSBs and incomplete HR at 24 h (Figure 8).

The study has limitations. Only one in vivo model was tested, no orthotopic CNS or lung model was included, and toxicity assessments were limited to MCF10A viability studies and body-weight monitoring in tumor-bearing mice. Although these findings suggest that the regimen was generally well tolerated, comprehensive hematologic analyses to assess potential myelosuppression [75] will be important to fully define its therapeutic window and support clinical translation. Another limitation is the relatively small number of animals used in the in vivo experiments, which may limit statistical power. Nevertheless, the observed treatment effects were consistent across cohorts and were supported by concordant findings from the in vitro studies.

In addition to HR and NHEJ, radiotherapy-induced DNA damage is processed by other DNA damage response pathways, including base excision repair (BER), which plays a critical role in repairing oxidative base lesions and single-strand breaks generated by ionizing radiation [76]. The potential contribution of BER to combined BUB1i- and CDK4/6i-mediated radiosensitization remains to be determined. Furthermore, treatment responses may be influenced by factors beyond intrinsic DNA repair capacity. Dietary antioxidants may modulate oxidative stress and radiation-induced ROS signaling [77,78], which may influence RT sensitivity, although their effects in the context of combined CDK4/6 and BUB1 inhibition remain to be determined. In addition, solid tumors contain both normoxic and hypoxic regions, and hypoxia is well known to influence DNA repair, genomic stability, and radiosensitivity, often contributing to therapeutic resistance compared with better-oxygenated tumor regions [79]. While hypoxia was not evaluated in the present study, future studies examining the impact of hypoxia may provide additional insights into treatment response.

Finally, the proposed mechanism is supported by DNA damage foci, reporter, and RPA phosphorylation analyses but not yet validated through formal epistasis or direct repair flux assays. Future studies should therefore incorporate time-resolved HR/NHEJ reporters, RAD51 and BRCA2 recruitment assays, RAD51 epistasis testing, and DNA fiber-based analysis of replication-coupled damage and ATR-CHK1 dependence.

Overall, these findings support co-targeting BUB1 and CDK4/6 as a radiosensitization strategy in GBM, lung cancer, and TNBC, particularly in Rb-intact settings.

## Figures and Tables

**Table 1:** Gene mutations in GBM, TNBC and LC cell lines.

Cell Line	*RB1*	RB1 Function Loss	*PTEN*	*TP53*
U251	WT	No	p.Glu242Valfs*15	p.Arg273His
A172	p.V852L	Unknown	Gene deletion	WT
U87	WT	No	c.209 + 1G > T	WT
9L	WT	No	Unknown	Mutated
SUM159	WT	No	WT	p.Val157_Arg158insLeu
MDA-MB-231	WT	No	WT	p.Arg280Lys
BT549	Null	Yes	p.Val275fs*1 (c.821delG)	p.Arg249Ser
HCC1937	p.Thr738_Arg775del38 (c.2212_2325del114)	Yes	Homozygous deletion	p.Arg306Ter (c.916C > T)
H1975	WT	No	WT	p.Arg273His
A549	WT	No	WT	WT
H596	Null	Yes	Homozygous deletion	p.Gly245Cys
MCF10A	WT	No	WT	WT

**Table 2:** Half-maximal inhibitory concentration (IC_50_) of BUB1i, ribociclib, and abemaciclib in glioma, TNBC, and lung cancer cell lines (µM).

Cell Lines	BUB1i	Ribociclib	Abemaciclib
U251	6.6	37	3.2
A172	4.9	48	1.6
U87	3.8	32	4
9L	6.2	>30	10
SUM159	2.5	52.1	1.3
MDAMB231	2.9	44.5	1.2
BT549	1.14	15.38	1.89
HCC1937	3.8	>50	3.8
H1975	2.6	>30	3.5
A549	1.8	14	3.3
H596	5.8	33	2.5
MCF10A	18	>60	45

## Data Availability

The datasets generated during and/or analyzed during the current study are available from the corresponding author on request.

## Supplementary Materials

The following supporting information can be downloaded at: https://www.mdpi.com/article/10.3390/biomedicines14091940/s1. Figure S1–S12; Table S1: Cell seeding numbers for clonogenic assay; Table S2: Experimental overview showing drug treatment and radiation timing, and endpoint readout.
