# Supplementary material for: BUB1 and CDK4/6 Dual Inhibition Increases Radiation Sensitivity in Glioblastoma, Lung Cancer, and Triple-Negative Breast Cancer
Source: Biomedicines. 2026 Aug 29;14(9):1940. doi: 10.3390/biomedicines14091940 (PMC13604186; doi:10.3390/biomedicines14091940)
Supplement: Supplementary file 1 [file biomedicines-14-01940-s001.zip › Supplementary tables.pdf]

Supplementary Table S1: Cell seeding numbers for clonogenic assay.

Cell lines	Cell numbers	Cell numbers	Cell numbers
	**0 Gy RT**	**2 Gy RT**	**4 Gy RT**
**Normal cell line**			
MCF10A	Mock (300), B250nM (400), R1μM (500), A100nM (500), B+R (600), B+A (600)	Mock (400), B250nM (600), R1μM (900), A100nM (900), B+R (1200), B+A (1200)	Mock (600), B250nM (900), R1μM (1800), A100nM (1800), B+R (2400), B+A (2400)
**GBM cell lines**			
U251	Mock (150), B250nM (200), R1μM (300), A100nM (400), B+R (450), B+A (450)	Mock (300), B250nM (400), R1μM (600), A100nM (700), B+R (900), B+A (900)	Mock (600), B250nM (800), R1μM (1200), A100nM (1400), B+R (2000), B+A (2000)
A172	Mock (300), B250nM (400), R750nM (500), A75nM (500), B+R (600), B+A (600)	Mock (600), B250nM (800), R750nM (1000), A75nM (1000), B+R (1200), B+A (1200)	Mock (1200), B250nM (1600), R750nM (2000), A75nM (2000), B+R (2400), B+A (2400)
U87	Mock (150), B250nM (200), R250nM (600), A25nM (600), B+R (1000), B+A (1000)	Mock (300), B250nM (400), R250nM (1200), A25nM (1200), B+R (2000), B+A (2000)	Mock (600), B250nM (800), R250nM (2400), A25nM (2400), B+R (4000), B+A (4000)
**TNBC cell lines**			
SUM159	Mock (200), B250nM (200), R1μM (200), A250nM (200), B+R (200), B+A (200)	Mock (300), B250nM (300), R1μM (300), A250nM (300), B+R (300), B+A (300)	Mock (450), B250nM (450), R1μM (450), A250nM (450), B+R (450), B+A (450)
MDA-MB-231	Mock (600), B250nM (600), R1μM (800), A100nM (800), B+R (1000), B+A (1000)	Mock (1200), B250nM (1500), R1μM (1800), A100nM (1800), B+R (2500), B+A (2500)	Mock (1800), B250nM (2000), R1μM (2500), A100nM (2500), B+R (3000), B+A (3000)
BT-549	Mock (200), B150nM (200), R1μM (200), A150nM (200), B+R (200), B+A (200)	Mock (350), B150nM (350), R1μM (350), A150nM (350), B+R (350), B+A (350)	Mock (450), B150nM (450), R1μM (450), A150nM (450), B+R (450), B+A (450)
HCC1397	Mock (1500), B150nM (1500), R500nM (1500), A150nM (1500), B+R (1500), B+A (1500)	Mock (3000), B150nM (3000), R500nM (3000), A150nM (3000), B+R (3000), B+A (3000)	Mock (4500), B150nM (4500), R500nM (4500), A150nM (4500), B+R (4500), B+A (4500)
SUM159 KO	Mock (200), R1.5μM (200), A250nM (200)	Mock (300), R1.5μM (300), A250nM (300)	Mock 450), R1.5μM (450), A250nM (450)
MDA-MB-231 KO	Mock (600), R1.5μM (600), A250nM (600)	Mock (1200), R1.5μM (1200), A250nM (1200)	Mock (1800), R1.5μM (1800), A250nM (1800)
**NSCLC cell lines**			
A549	Mock (300), B250nM (350), R1μM (450), A100nM (450), B+R (550), B+A (550)	Mock (600), B250nM (700), R1μM (900), A100nM (900), B+R (1000), B+A (1000)	Mock (1200), B250nM (1400), R1μM (1800), A100nM (1800), B+R (2000), B+A (2000)
H1975	Mock (250), B250nM (400), R1μM (400), A100nM (400), B+R (400), B+A (400)	Mock (500), B250nM (750), R1μM (750), A100nM (750), B+R (750), B+A (750)	Mock (1000), B250nM (1500), R1μM (1500), A100nM (1500), B+R (1500), B+A (1500)
H596	Mock (300), B250nM (400), R750nM (500), A75nM (500), B+R (600), B+A (600)	Mock (600), B250nM (800), R750nM (1000), A75nM (1000), B+R (1200), B+A (1200)	Mock (1200), B250nM (1600), R750nM (2000), A75nM (2000), B+R (2400), B+A (2400)
H1975 KO	Mock (250), R1μM (400), A150nM (400)	Mock (500), R1μM (750), A150nM (750)	Mock (1000), R1μM (1500), A150nM (1500)
A549 KO	Mock (300), R1μM (350), A150nM (350)	Mock (600), R1μM (700), A150nM (700)	Mock (1200), R1μM (1300), A150nM (1300)

Supplementary Table S2: Experimental overview showing drug treatment and radiation timing, and endpoint readout

Assay	Treatment Schedule	Irradiation Timing	Endpoint Readout
**Cell viability (AlamarBlue)**	72 h	None	Cell viability, IC_{50}
**Clonogenic survival**	7-14 days	RT delivered 1 h or 24 h after drug treatment	Plating efficiency (PE), rER
**γH2AX, RAD51, RPA, and BrdU foci assays**	2 h and 24 h	RT delivered 1 h after drug treatment	DNA damage repair intermediates
**DNA damage reporter assay**	48 h	RT delivered 1 h after drug treatment	HR and NHEJ repair activity
**qRT-PCR**	72 h	RT delivered 1 h after drug treatment	Relative gene expression
**Cell cycle analysis**	24 h	RT delivered 1 h after drug treatment	Cell cycle distribution
**Comet assay**	24 h	RT delivered 1 h after drug treatment	Olive tail moment, DNA damage
**Micronucleus assay**	24 h	RT delivered 1 h after drug treatment	Micronucleus
**In vivo tumor studies**	Drugs: 5 days x 3 weeks	Fractionated RT (5 Gy × 3)	Tumor growth inhibition
