# Supplementary material for: BUB1 and CDK4/6 Dual Inhibition Increases Radiation Sensitivity in Glioblastoma, Lung Cancer, and Triple-Negative Breast Cancer
Source: Biomedicines. 2026 Aug 29;14(9):1940. doi: 10.3390/biomedicines14091940 (PMC13604186; doi:10.3390/biomedicines14091940)
Supplement: Supplementary file 1 [file biomedicines-14-01940-s001.zip › Supplementary figures.pdf]

## Supplementary Figure S1

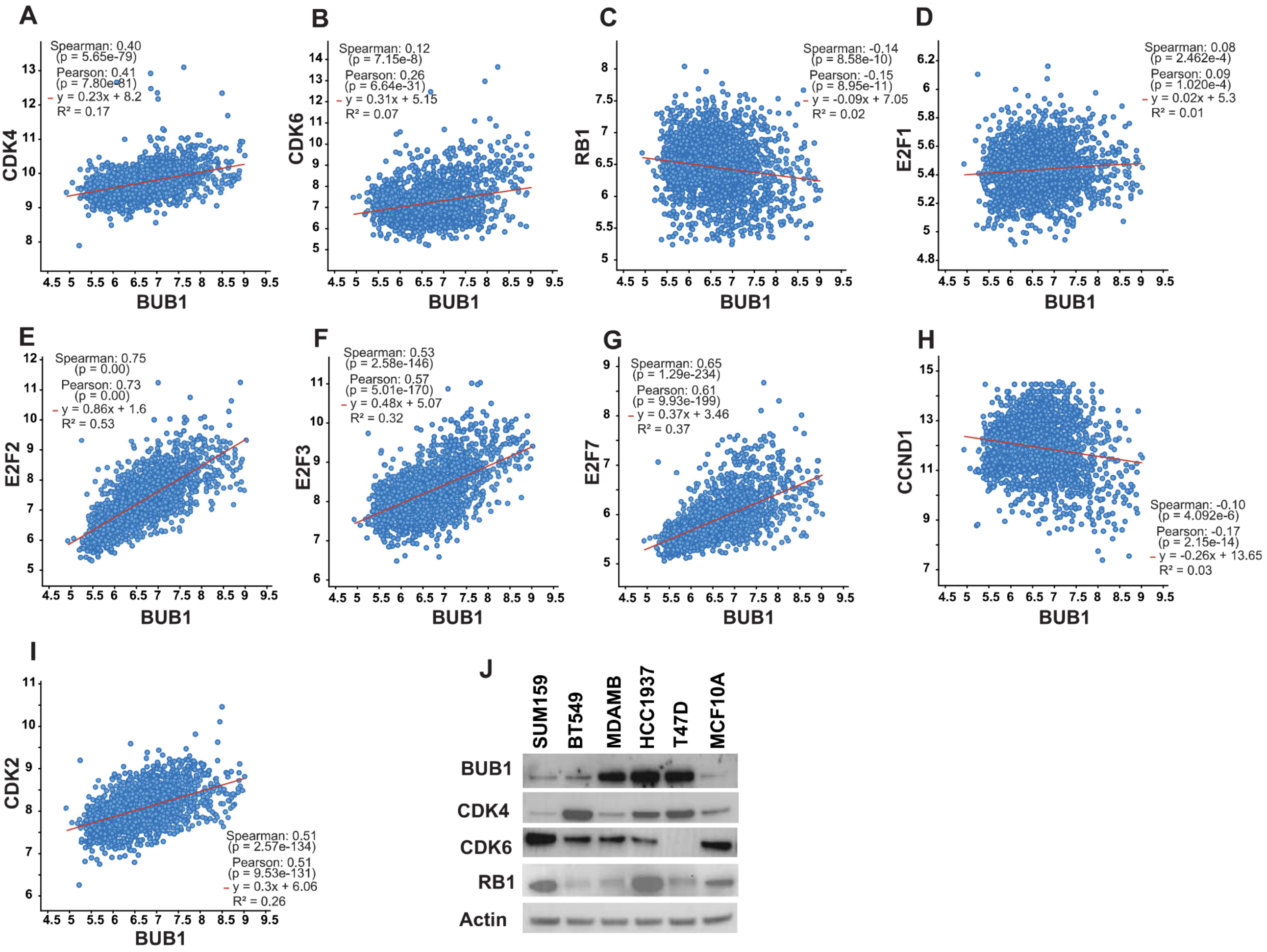

Supplementary Figure S2

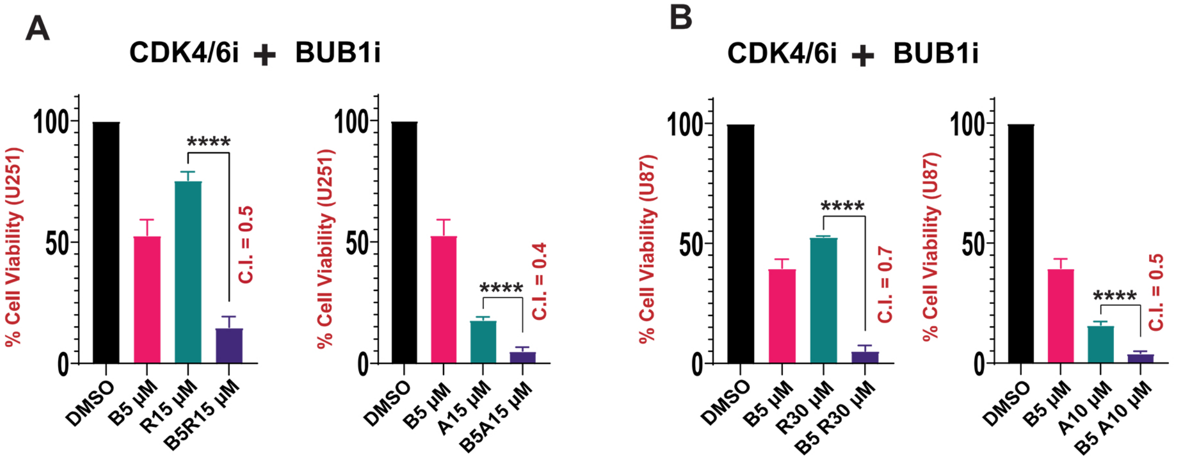

Supplementary Figure S3

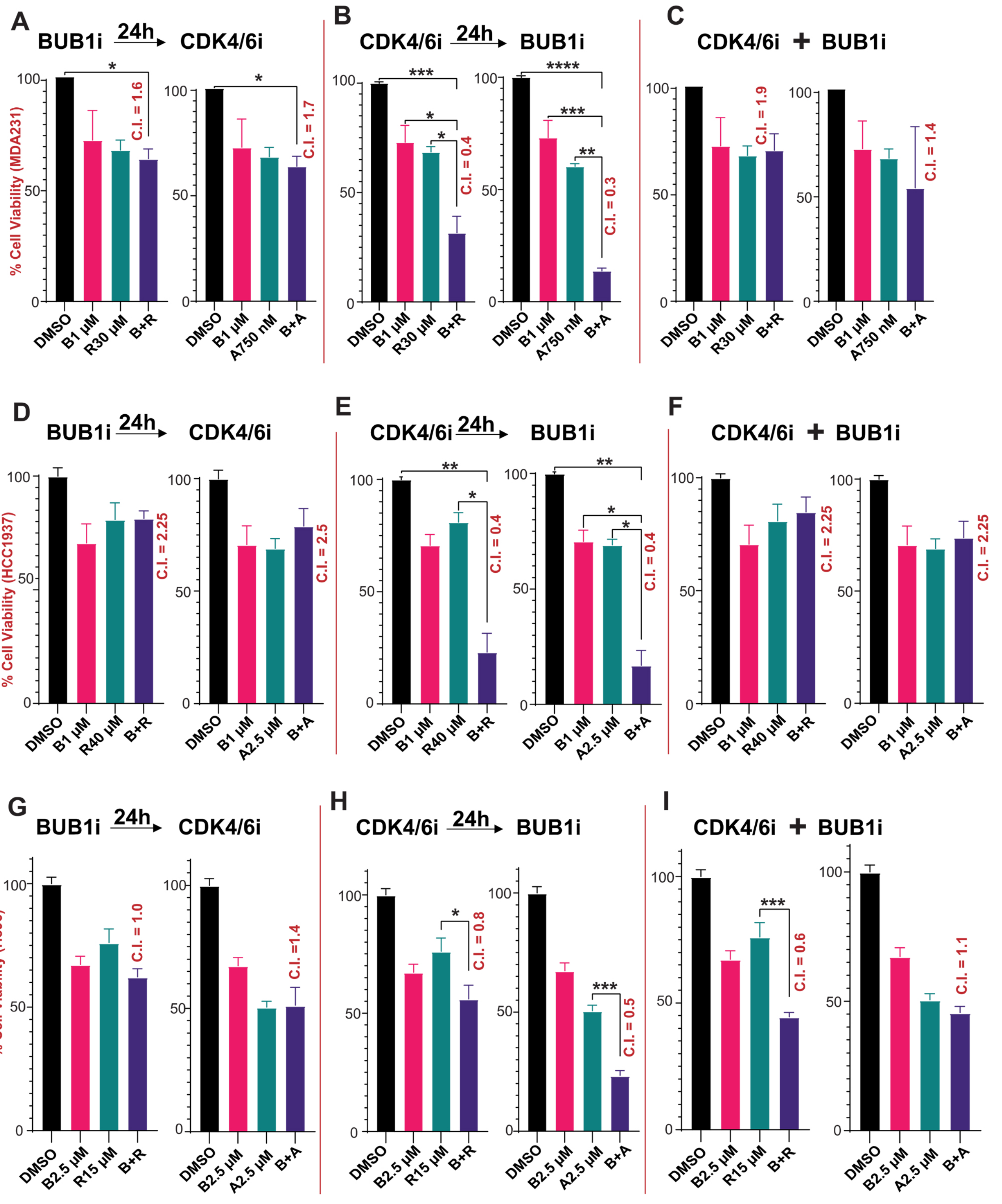

## Supplementary Figure S4

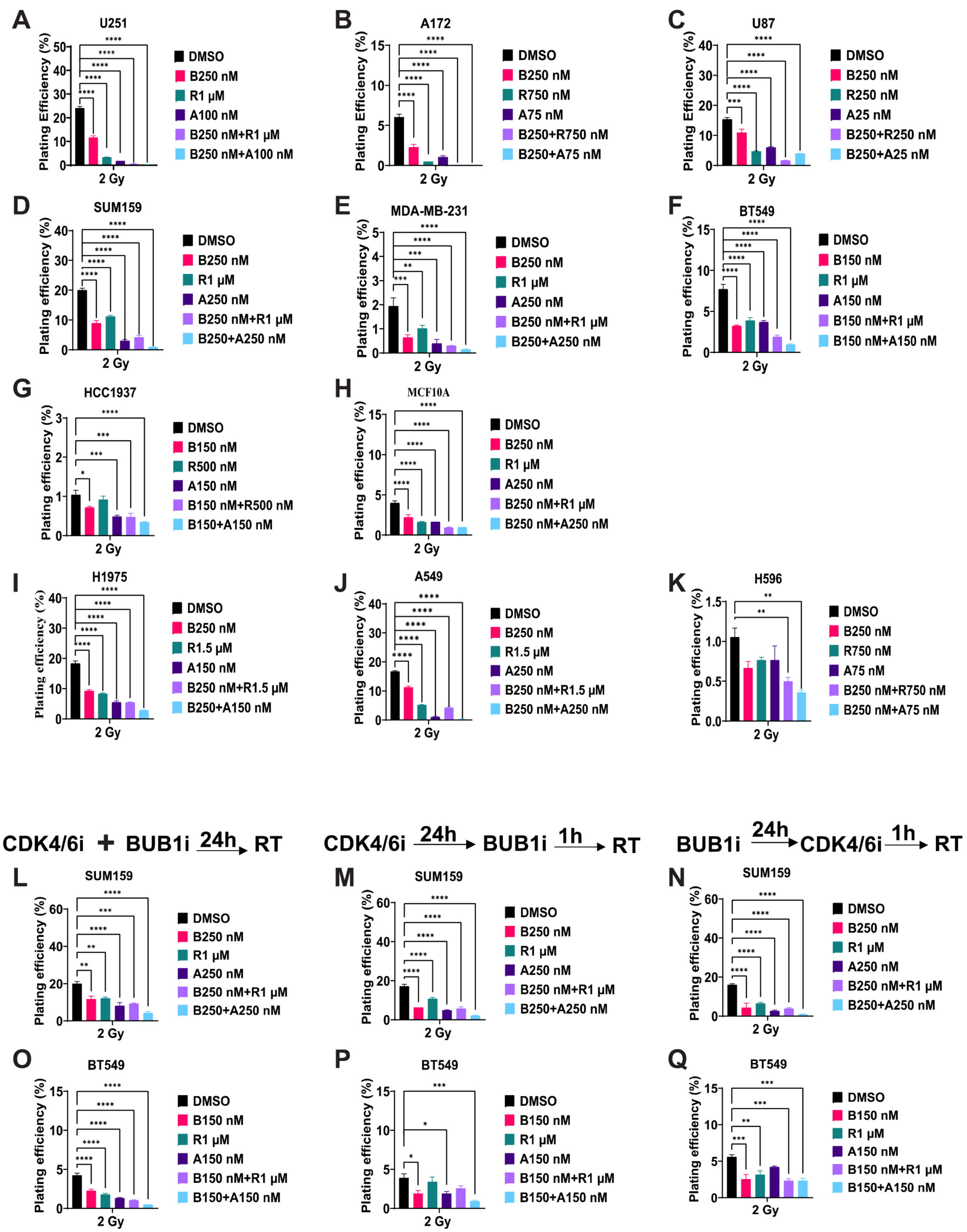

# Supplementary Figure S5

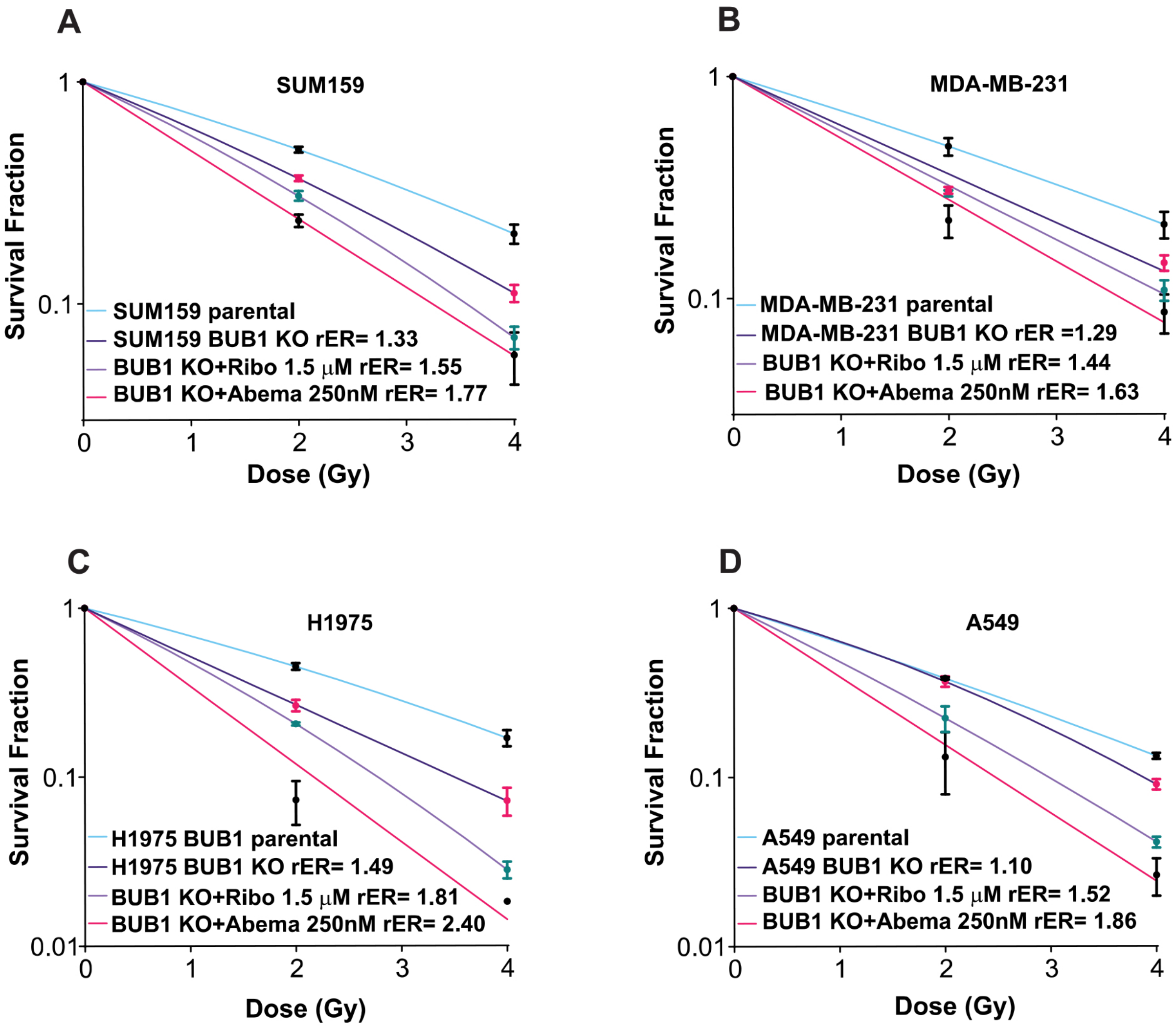

**Supplementary Figure S6**

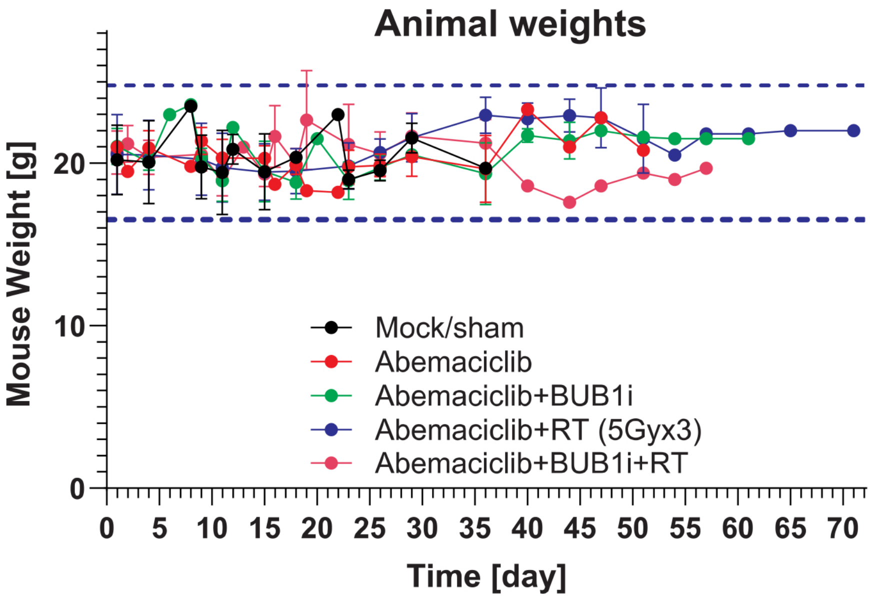

# Supplementary Figure S7

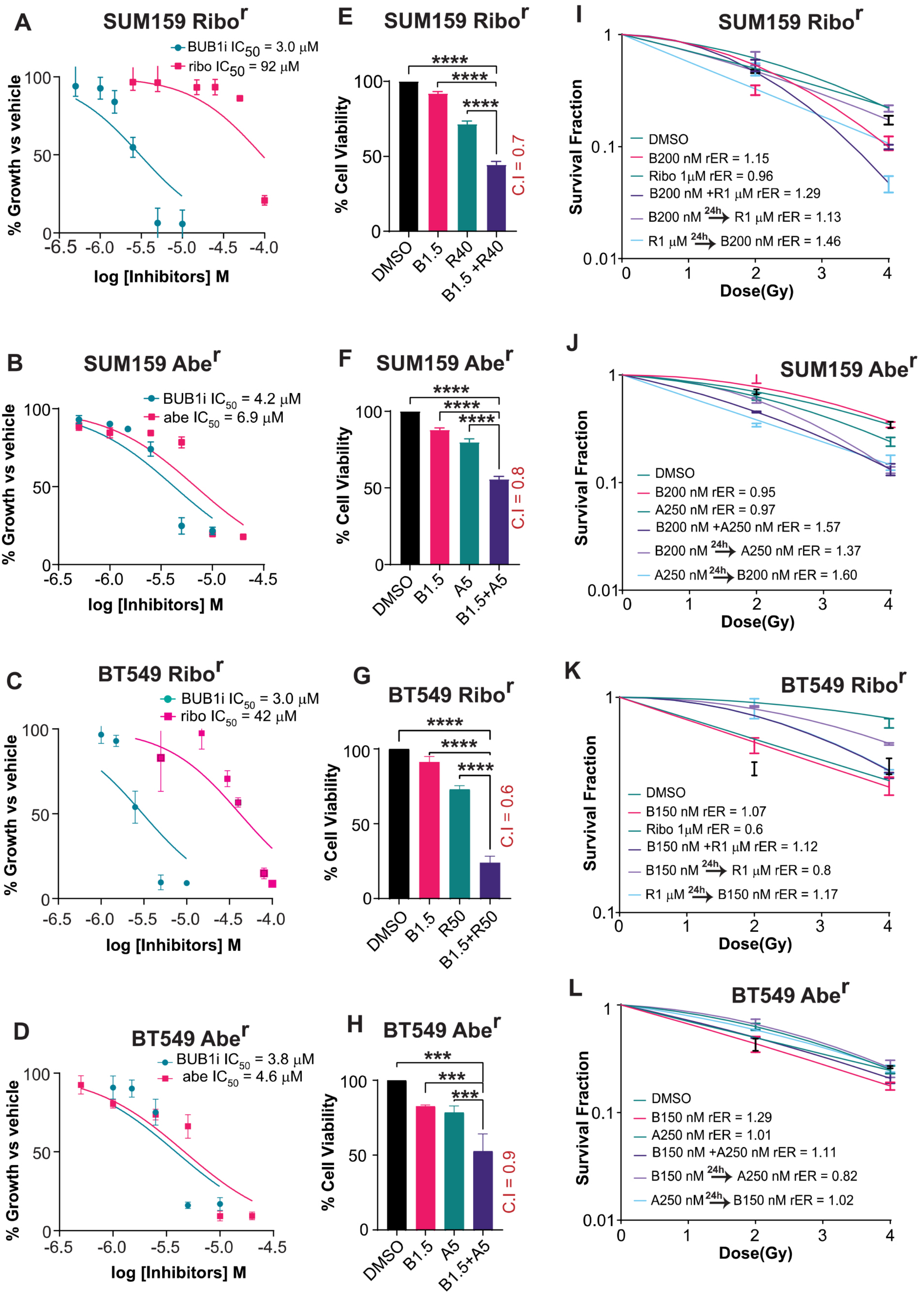

## Supplementary Figure S8

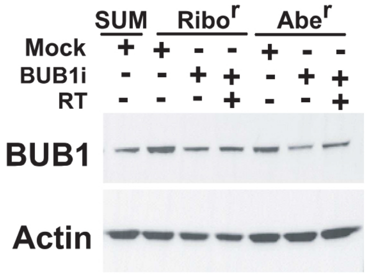

## Supplementary Figure S9

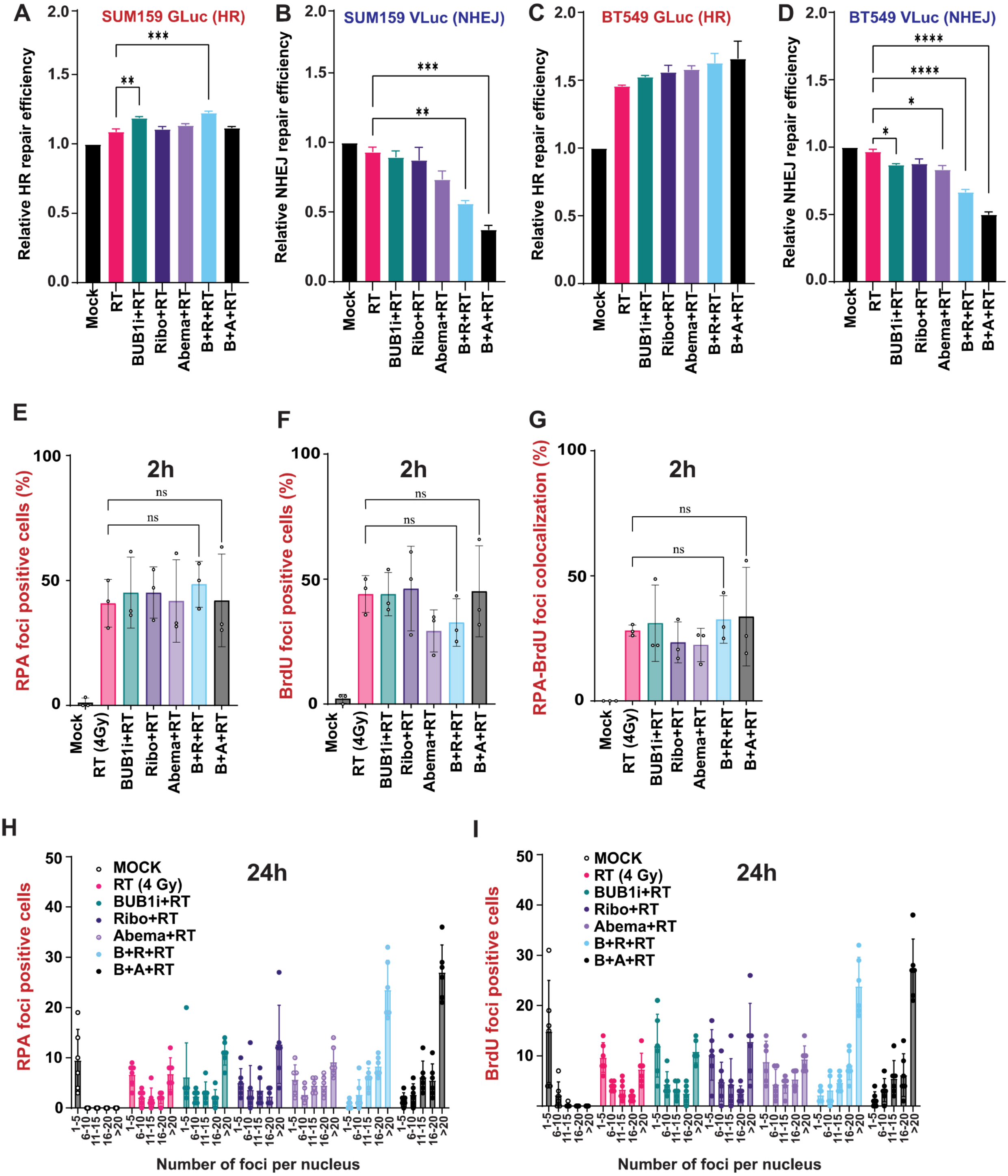

## Supplementary Figure S10

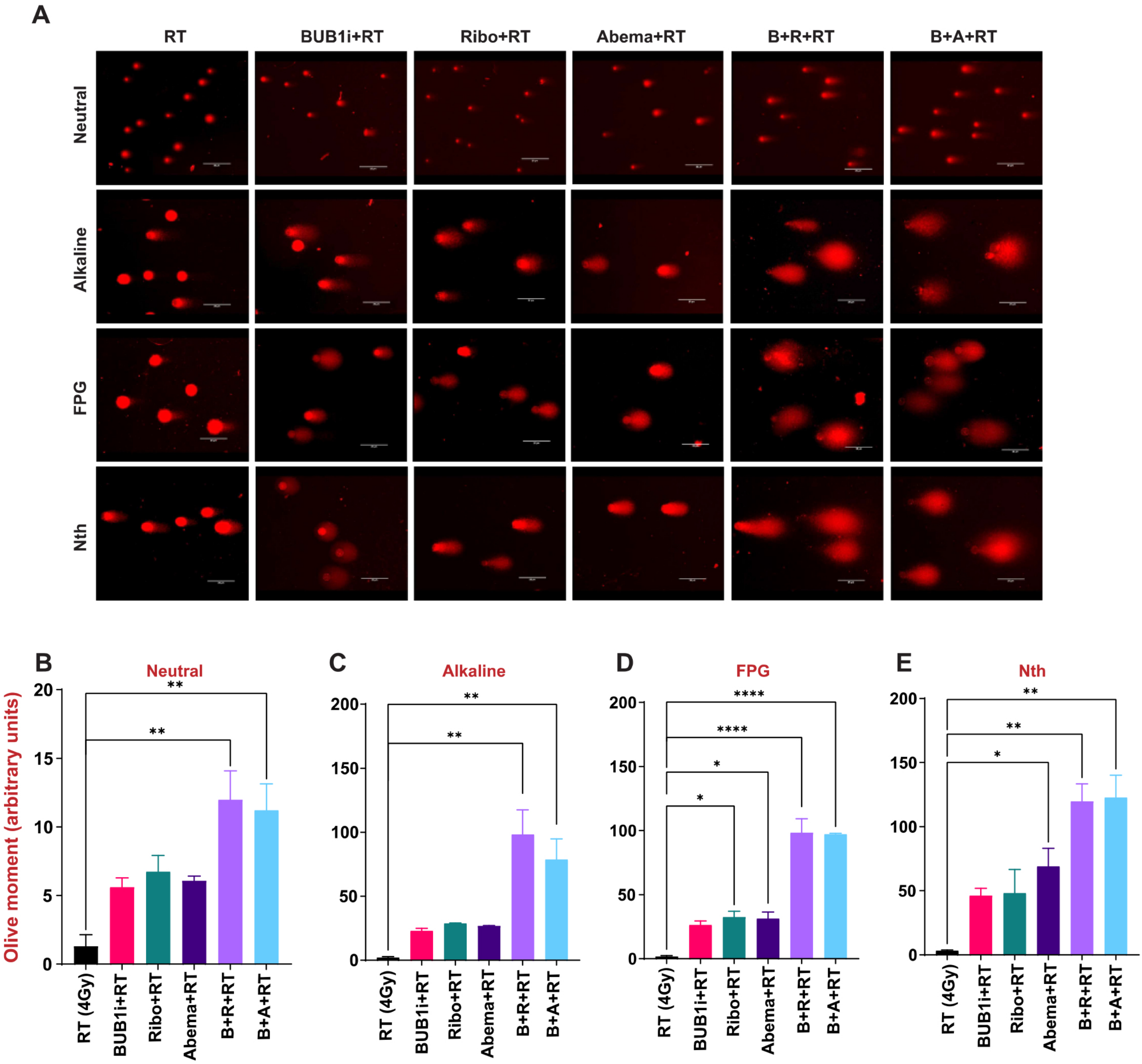

**Supplementary Figure S11**

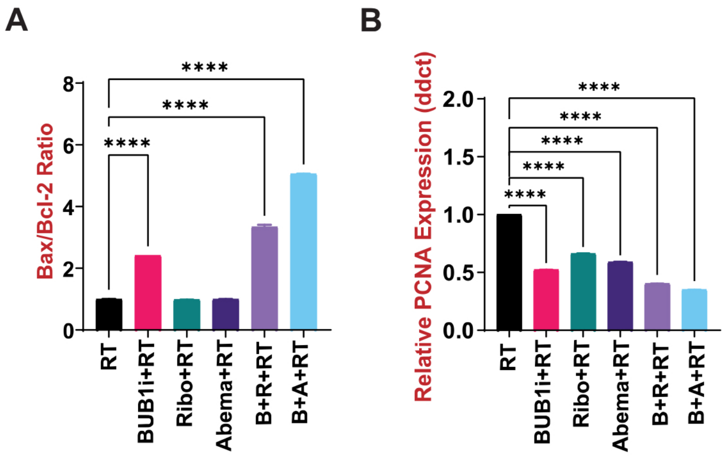

## Supplementary Figure S12

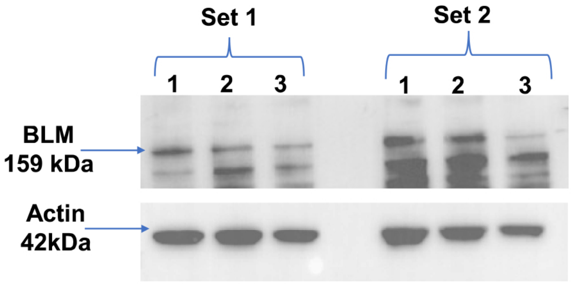


Lanes

1. SUM159
2. SUM159+NT siRNA
3. SUM159+BLM siRNA
